# Fast Episodes of West-Mediterranean-Tyrrhenian Oceanic Opening and Revisited Relations with Tectonic Setting

**DOI:** 10.1038/srep14271

**Published:** 2015-09-22

**Authors:** Carlo Savelli

**Affiliations:** 1 Consiglio Nazionale delle Ricerche, Istituto di Scienze del Mare, via P. Gobetti, 101, 40129, Bologna, Italy

## Abstract

Extension and calc-alkaline volcanism of the submerged orogen of alpine age (OAA) initiated in Early Oligocene (~33/32 Ma) and reached the stage of oceanic opening in Early-Miocene (Burdigalian), Late-Miocene and Late-Pliocene. In the Burdigalian (~20–16 Ma) period of widespread volcanism of calcalkaline type on the margins of oceanic domain, seafloor spreading originated the deep basins of north Algeria (western part of OAA) and Sardinia/Provence (European margin). Conversely, when conjugate margins’ volcanism has been absent or scarce seafloor spreading formed the plains Vavilov (7.5–6.3 Ma) and Marsili (1.87–1.67 Ma) within OAA eastern part (Tyrrhenian Sea). The contrast between occurrence and lack of margin’s igneous activity probably implies the diversity of the geotectonic setting at the times of oceanization. It appears that the Burdigalian calcalkaline volcanism on the continental margins developed in the absence of subduction. The WNW-directed subduction of African plate probably commenced at ~16/15 Ma (waning Burdigalian seafloor spreading) after ~18/16 Ma of rifting. Space-time features indicate that calcalkaline volcanism is not linked only to subduction. From this view, temporal gap would exist between the steep subduction beneath the Apennines and the previous, flat-type plunge of European plate with opposite direction producing the OAA accretion and double vergence.

The West-Mediterranean-Tyrrhenian oceanic opening has gradually become recognized as the consequence of interactions between the European and African plates, and of subduction polarity reversal. From the Late Cretaceous to the Eocene/Oligocene boundary, the SE-directed (Alpine-type) flat-slab’s subduction of the European plate, originated the Mediterranean’s submerged orogen of Alpine age (OAA)^1^,^2^,^3^. The convergence tectonics of the OAA has terminated by the flip of subduction vergence. The OAA, from Alpine Corsica to the Betic Cordillera (Fig. 1; Fig. 2A) was recognized as the westernmost branch of the western Alps related to the pre-Oligocene closure of the Mesozoic Alpine Tethys^4^,^5^,^6^. Despite its young age, the tectonic setting of the region under examination has been subject to various interpretations (see Supplementary Info S1). However the largely accepted idea considers that, since the Oligocene, extension seafloor spreading and calc-alkaline volcanism developed entirely above the WNW-directed, steep-slab-type (Island-arc-type) subduction of African plate and at the rear of the Apennine wedging^3^,^7^. From this point of view, the West Mediterranean and the Tyrrhenian represent two distinct back-arc regions, formed however above the same subduction which was retreating eastward due to slab rollback and passive sinking.

In Earth Sciences, magmatism of the calc-alkaline type is generally viewed as a result of partial melting of sources, which have undergone metasomatic modification via chemical recycling of subducted (mainly upper) crustal material. However, the temporal interval between subduction and igneous manifestations is matter of debate^8^,^9^,^10^,^11^. The pervasive presence of calc-alkaline volcanism above the zones of Recent plate convergence is probably at the origin of the tenet that melt uprising and metasomatism overlapped in time with subduction in the geological past, too. On the other hand, the geological record suggests that metasomatism has probably taken place earlier than the calc-alkaline eruptive activity^12^,^13^,^14^. This work, based on comparative examination of the distribution of continental margin volcanism at the times of oceanization, recognizes and tentatively quantifies the temporal gap between *old* and *new* (*reversed*) polarity of subduction. The gap may be useful to unravel the tectonic setting linked to calc-alkaline volcanism of the past 33 Ma. Igneous geochemistry data, from literature on the study area, is listed in the Supplementary Datasets S4, S6, it being a non-fundamental issue. Some of the examined geochronology data could be incorrect because of the diverse quality of the analysed material, and diversity of laboratories and analytical methods. However, dubious (few) ages can be tentatively pointed out, based on the large data set currently under examination and plausible temporal correlation.

Extension and magmatism commenced in Early Oligocene and reached the stage of oceanization by ~20–16 Ma (Burdigalian). Early Miocene sea-floor spreading formed the oceanic crust flooring of the north Algeria and of Sardinia-Provence deep plains. Overall, basin formation is linked to rotation of small continental blocks (Fig. 1)^15^,^16^,^17^. The Tyrrhenian Sea’s deep plain came into existence (Figs 1 and 2b) only after the Burdigalian age’s counter-clockwise rotation of the eastern part of the OAA which, together with its Hercynian foreland of Corsica-Sardinia drifted away from the European margin (SE France). The Sardinia-Provence and North Algeria basins are separated by the major transcurrent structure known as the “north Balearic fracture zone” or the “Paul Fallot transform fault” which dates back to plate interactions of the Hercynian orogeny^4^,^16^. This structure, extending from the Catalan volcanic zone to the magmatic island of La Galite (Tunisian offshore) - hereafter called “Catalan-Tunisian fracture zone” (CTFZ) - separates the western segment of the Mediterranean OAA and the European margin from that to the east. In particular, the fracture zone divided Sardinia-Provence deep plain (Hercynian European margin) from the western segment of the OAA (north Algerian basin), and this from the eastern one. Another major lineament running along the 41° parallel^1^,^17^ separates the northern Tyrrhenian’s thinned continental crust from the oceanic crust to the south. The two areas are rimmed by the Apennines, which rotated counter-clockwise and clockwise, respectively to the north and south.

Volcanoclastic rocks showing calc-alkaline nature, are widespread among the allochthonous sediments of the Apennines (Fig. 3; Supplementary Info S3 and Datasets S5, S6). If they have been produced from lost emission centres, which were originally sited in the Tyrrhenian OAA, their space-time distribution can also be meaningful for the reconstruction of the link between calc-alkaline volcanism and tectonic setting. For the first time, allochthonous and “*in-situ*” volcanics were jointly considered.

## The episodes

This chapter examines the episodes of fast sea-floor spreading, and the ditribution of continental margin magmatic rocks which erupted in the course of the first episode (~20-16 Ma). In the Supplementary Material are discussed: (i) magmatics that are not coeval with the oceanization events of the Late Miocene and Late Pliocene, (ii) the volcanogenic allochthons of calc-alkaline nature of the Apennines and (iii) the buried Apenninic volcano from site 13/Fig. 2B.

### Fast sea-floor spreading

#### Burdigalian (~20–16 Ma)

**Figure 1:**
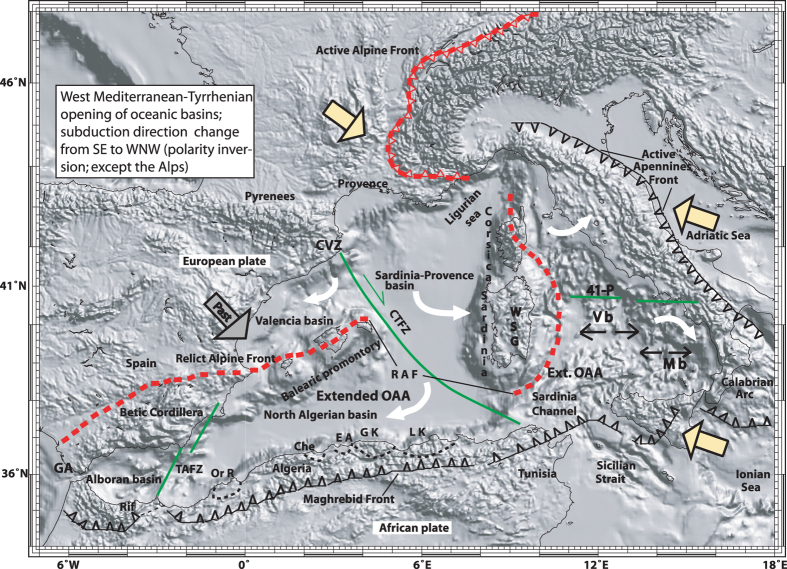
Relief-shaded image of the west Mediterranean-Tyrrhenian area showing the Recent convergence fronts of the Maghreb-Apennines and the Alps (s.s.). The red dashed line indicates the proposed front of past (pre-Oligocene) subduction of European plate beneath the orogen of Alpine age (**OAA**). The large gray arrow points to past SE-directed subduction plunge, and the yellow arrows indicate active subduction with SE (Alpine) and WNW (Apennines) polarity. The white arrows show the counter-clockwise rotation of Corsica-Sardinia and north Apennines, and the clockwise rotation of the Algeria margin and south Apennines. Legend: the oceanic basins of Sardinia-Provence (**Sa-Pr b**), Vavilov (**V b)**, Marsili (**M b)**. **CTFZ** = Catalan -Tunisian fracture zone separating the west segment of European plate and extended OAA from that to the east (see text for discussion); **CVZ** = Catalan volcanic zone; **GA** = Gibraltar arc; **RAF** = Relict Alpine Front; **TASZ** = Trans-Alboran shear zone; **WSG** = west Sardinia graben. The Algerian Maghreb magmatic sites: **Or R** = Oranois; **Che** = Cherchell; **EA** = East of Algiers; **GK** = Great Kabylia; **LK** = Little Kabylia (see also Supplementary dataset S4); **41-P** = 41° parallel lineament. The figure was created by the author with the use of plotmap software^41^.

**Figure 2:**
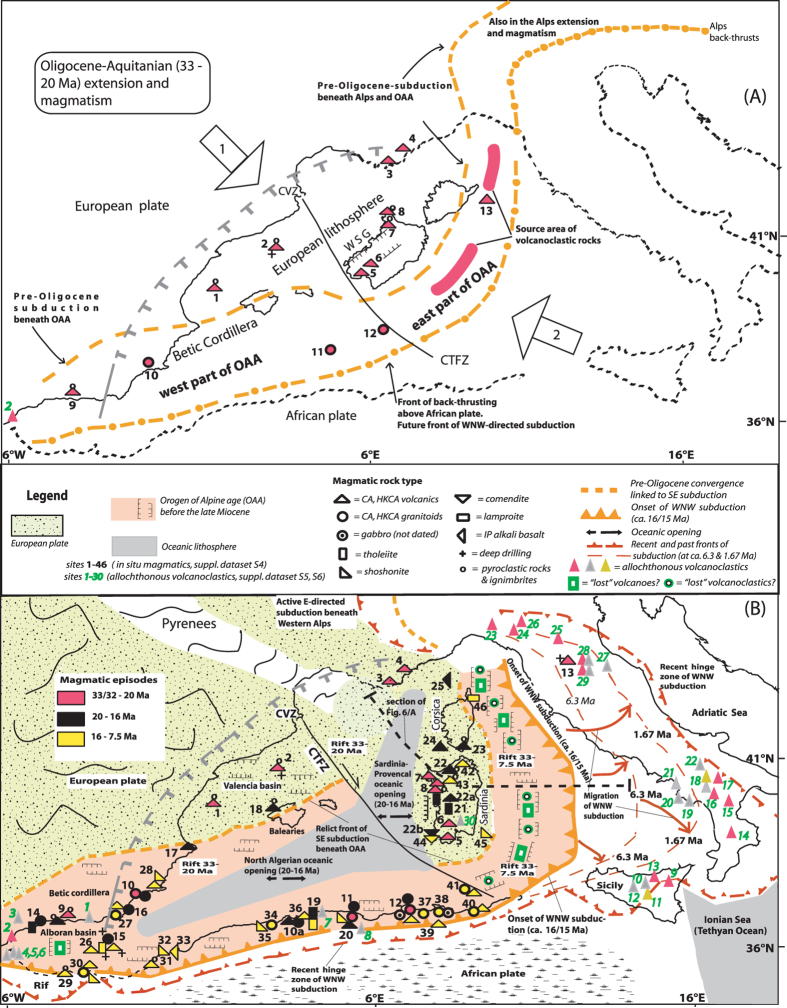
Distribution of “*in situ*” magmatics of the west Mediterranean, and allochthonous volcanoclastics of the Apennines. (**A)** Oligocene-Aquitanian (between ~33/32 and ~20 Ma): intrusives (red circle), volcanics (red triangle) and allochthonous volcanoclastics (elongated red triangle without rim shows presumed position; green numbers in *italics;* see Suppl. dataset S4). Site 13: buried andesite volcano of the early Oligocene. White arrows: 1 = pre-Oligocene (>33 Ma), SE-directed subduction beneath the orogen of Alpine Age (OAA); 2 = Post-Burdigalian (<16 Ma), WNW-directed subduction of African lithosphere; this reconstruction considers that subduction was absent in the Oligocene - Burdigalian time. (**B**) Burdigalian (~20–16 Ma): intrusives (black circle), volcanics (black triangle) and allochthonous volcanoclastics (elongated gray triangle); Langhian-Tortonian (between ~16 and 7.5 Ma): intrusives (yellow circle), volcanics (black rimmed yellow triangle) and allochthonous volcanoclastics (elongated yellow triangle). In the Burdigalian, tholeiitic lavas accompanied the calc-alkaline magmatism of andesitic and silicic type as the continental rifting reached the stage of oceanic spreading in the Sardinia-Provence and north Algerian basins respectively on the east and on the west of the Catalan-Tunisian fracture zone (CTFZ). The scheme shows the proposed position of the WNW subduction hinge zone at ~6.3 (Vavilov opening) and ~1.67 Ma (Marsili opening), the Apennine outcrops of the allochthonous volcanoclastic rocks, and the hypothetical sites of “lost” volcanic edifices and adjacent sedimentary lows which, according to the geodynamic interpretation (see *Guerrera et al.*, 1998, and *Cibin et al.,* 2001 in the Suppl. info), were originally sited in the Tyrrhenian OAA (offshore Sardinia-Corsica). Dotted line: the lithosphere-asthenosphere section of Fig. 6A. The figure was created by the author with the use of plotmap software^**41**^.

Between ~20 and 16 Ma, the oceanization of European lithosphere and submerged western part of OAA originated, respectively, the Sardinia-Provence and north Algeria deep basins (Figs 1 and 2). The Burdigalian timing of the two openings has been ascertained only from the geology of continental margin as deep drilling data from the basaltic crust are not available. The origin of Sardinia-Provence basin is linked to counter-clockwise rotation of Corsica-Sardinia; the age of rotation has been determined mainly from the combination of paleomagnetic and geochronological data of volcanic rocks from the magma-rich Corsica- Sardinian margin^18^,^19^,^20^. Temporal overlap between the Sardinia-Provence and north Algeria basin opening has been considered by various authors^1^,^3^,^16^. 17.8, 17.4 Ma are reported^21^ for peridotite emplacement at the Edough granitoid Massif of Little Kabylia (eastern Maghrebides; Fig. 2; site 12). The authors propose temporal overlap between the onshore tectonics and seafloor spreading of the north Algeria basin. Along the coastal area of Algeria, granitoids, and andesites and dacites erupted at ~16-15 Ma^22^. The authors consider that such short-lived intense magmatism ought to be connected with slab break-off, and the last-stage of seafloor spreading in the Algerian offshore.

#### Late Miocene (~7.5–6.3 Ma)

**Figure 3:**
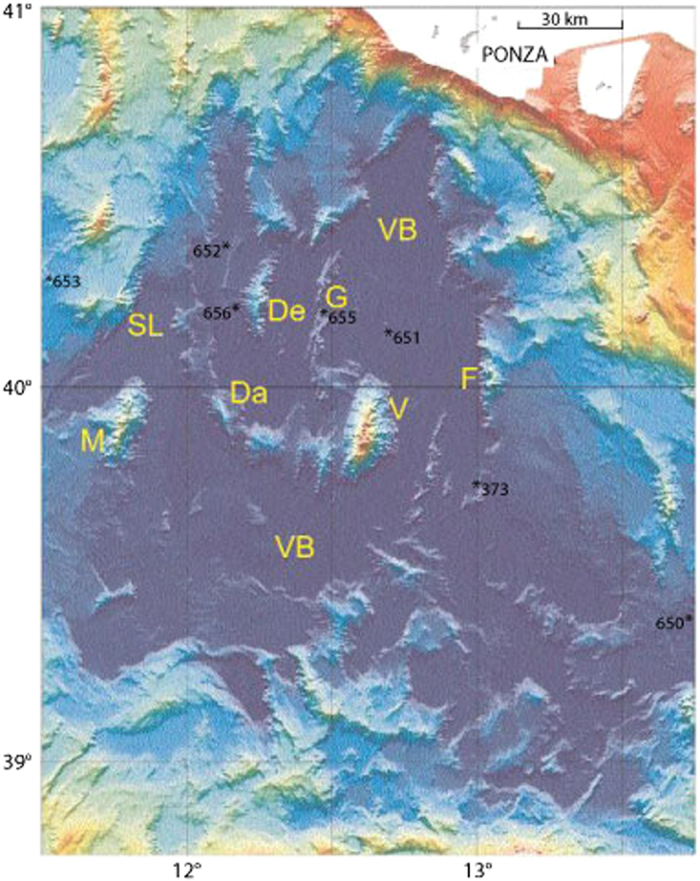
Multibeam seafloor morphology of the Vavilov bathyal plain; illumination from the NW. Main morphological elements are: the large volcanoes Vavilov (V) and Magnaghi (M), the Gortani (G) and D’Ancona (Da) ridges, the De Marchi (De) and Flavio Gioia (F) tilted blocks and the Selli Line (SL) fault. Refer to text for discussion of salient features of the DSDP and ODP drillsites; the site of ODP 654 drilling is placed 65 km (35 nm) to the WNW of site 653. From the “Memorie Descrittive della Carta Geologica d’Italia, Vol. LXIV (2004) ISPRA”; reproduced with permission of ISPRA. http://www.isprambiente.gov.it/en/publications/technical-periodicals/descriptive-memories-of-the-geological-map-of/from-seafloor-to-deep-mantle-architecture-of-the.

Deep drilling data, data of basement lithology^23^,^24^ and multibeam mapping^25^ are essential for understanding the complex evolution of Tyrrhenian seafloor. However, the nonexistence of the typical lineated magnetic anomalies is at a disadvantage^26^. In the northern part of the Vavilov bathyal plain (VB; 3400–3600 m bsl; Fig. 3), two seamounts represent stretched relics of OAA. The western De Marchi (De) and the Flavio Gioia (F) seamount to the east show N-S trend and elevation of 1200 m above the plain. The NE-SW Selli lineament (SL) is an important morphological feature located between the bathyal area and the passive margin offshore Sardinia. The former area belongs to the stretched OAA and the latter to the Hercynian lithosphere of rotated Corsica-Sardinia block. The SL is interpreted^24^ as the sea-floor expression of low-angle, east-dipping detachment faulting of continental crust which soles in the upper mantle beneath the Magnaghi/Vavilov basin. The N-S oriented, 40 km long Gortani ridge (G) with elevation of ca. 300/400 m is positioned between the De Marchi and Flavio Gioia. In G, beneath 80-m-thick sediments of late Pliocene – Quaternary age, the ODP well- 655^23^ drilled basalts of MORB (Mid Ocean Ridge Basalt) type. To the east of (G) and north of Vavilov seamount (V), ODP well-651 is floored with mantle peridotite^23^. In the sourthern part of the Vavilov plain between the big volcanoes Magnaghi (M) and (V), the arcuate D’Ancona (Da) ridge initiates between the SL and De Marchi with elevation of 200–400 m and sediment cover up to 250 m. This structure may reflect the complex nature of oceanization processes within the stretched bathyal relics of OAA.

**Figure 4:**
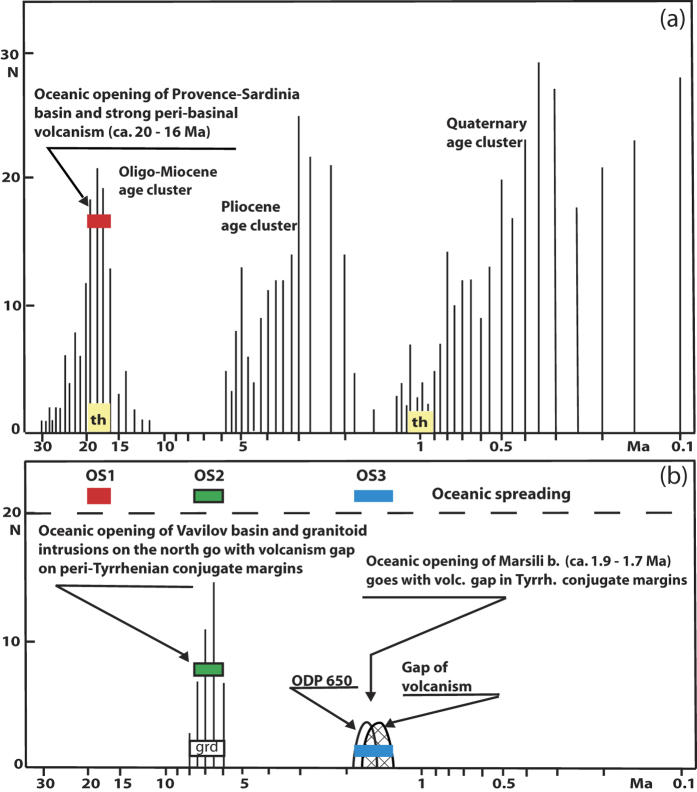
Histogram of age data (number of ages vs age data) of magmatics from the Provence-Corsica-Sardinia-Tyrrhenian-Peninsular Italy area since the Oligocene. (**a**) = the age clusters of the Oligocene -mid Miocene (~33/32–12 Ma), Pliocene (~5.4–1.8 Ma) and Quaternary (~1.2–0 Ma; see Fig. 5 and text); (**b**) = the episodes of oceanic spreading (OS) which formed the basins of Sardinia-Provence, Vavilov and Marsili. With respect to the basins’s conjugate margins, volcanic activity has been abundant during OS1, and absent or scarce during OS2 and OS3. Legend: th = tholeiites; grd = granitoids. The Burdigalian-age tholeiites are coeval with peak volcanism, and seafloor tholeiites of Aeolian arc (~1 Ma) pre-date the acme of volcanism (~0.2 Ma). The figure was created by the author.

In the eastern rim of Vavilov bathyal plain, 3507 m b. s. f. (Fig. 4) DSDP well-373 drilled 190 meter of basalt flow and breccia below 280-m-thick marls of early Pliocene-Quaternary age. The rocks exhibit MORB-like composition.Six whole rock K/Ar determinations between 7.5 and 6.3 +/−0.8 Ma (Late Tortonian/Early Messinian) indicate that the oldest-known basaltic crust of Tyrrhenian Sea formed before the global event called the “Mediterranean salinity crisis”. In fact, the evaporitic sedimentation was initiated at 5.96 Ma^27^, after the start of Messinian (~7.25 Ma)^28^, and ended at 5.33 Ma (start of the Pliocene). Evaporites are most likely not present in the lower Sardinian margin^23^. The authors, based on the evaporite occurrence in the upper Sardinian margin and on absence or scarcity in the lower one, consider that seafloor depth might have been diverse during the evaporitic episode. In this view, the shallow seafloor of lower Sardinian margin and adjacent Vavilov plain, too, might have impeded Atlantic-water-inflow in the sufficient amount to precipitate evapotitic gypsum.

**Figure 5:**
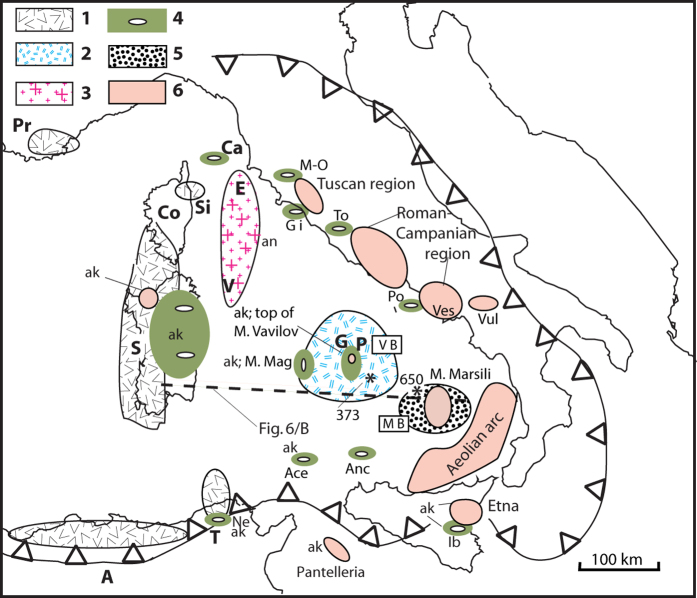
Distribution scheme showing the “*in situ*” magmatic rocks of the Tyrrhenian region and surroundings. Legend: **1** = calc-alkaline volcanics erupted between ~33/32 and 12 Ma; **2** = oceanic spreading of Vavilov bathyal plain (~7.5–6.3 Ma; DSDP well-373, see *); **3** = post-orogenic granitoids of the north Tyrrhenian (~8 to 6 Ma, late Miocene); **4** = magmatic rocks erupted between ~5 and 2 Ma (Pliocene); **5** = oceanic spreading of Marsili bathyal plain (~1.87–1.67 Ma; ODP well-650, see *); **6** = <1.2 Ma volcanics (Late Quaternary). ak = magmatic rocks exhibiting alkaline (OIB) character, and Pliocene or Quaternary age. In Capraia island, Nefza area and Iblei mounts are present also volcanics of late Miocene age. Abbreviations: A = Algeria, Ace = seamount Aceste, Anc = seamount Anchise, Ca = Capraia island, Co = Corsica, E = Elba island, G = Gortani ridge (ODP site 655), Gi = Giglio island, M-O = Montecatini V/C - Orciatico, M. Mag = seamount Magnaghi, MB = Marsili basin, Ne = Nefza area, P = ocean-floor peridotite (ODP site 651), Po = Ponza and Palmarola islands, Pr = Provence, S = Sardinia, Si = Sisco; T = Tunisia, To = Tolfa, V = Vercelli seamount, VB = Vavilov basin, Ves = Vesuvius - Campi Flegrei, Vul = M. Vulture. Dotted line shows the position of lithosphere-asthenosphere section of Fig. 6B. The figure was created by the author with the use of plotmap software^**41**^.

The interaction among faulting and magmatism played a significant role in the development of Tyrrhenian’s seafloor spreading. Seismic stratigraphy^24^ indicates east-dipping low-angle detachment faults producing Late-Tortonian/Early-Messinian strong extensional deformation on the continental margin offshore Sardinia. The E-W oriented hyperextension of the southern part of Tyrrhenian Sea appears to be coeval with the punctiform MORB-type volcanism of DSDP well-373. At about the same time span, granitoids erupted in the northern part (~8/6 Ma; Figs 4 and 5). The intrusive rocks are distributed from the southern Vercelli seamount and Etruschi ridge to the subaerial outcrops of the islands of Montecristo and Elba to the north^17^,^29^. Overall, localized basalt volcanism, not-lineated and low-standing, combined with strong extensional deformation and mantle peridotite exposure (DSDP well-651) would characterize the Vavilov’s seafloor speading, thus providing useful constraints for better understanding the early stage of the N-E Atlantic opening^30^.

#### Late Pliocene (~1.87–1.67 Ma)

Below 600-m-thick ooze sediment, ~1.87 to 1.67 Ma old basalt flows showing MORB-type to transitional composition erupted on the floor of Marsili plain western rim^23^ (ODP well-650; Fig. S1b of Supplementary Info S1). Sediment at direct contact with the basalt shows latemost Pliocene age. The round-shaped positive magnetic anomaly of the eruption area has been attributed to the Olduvai chron (1.87–1.67 Ma)^23^,^26^. The gap of volcanic activity of the Tyrrhenian’s conjugate margins partly overlaps the seafloor spreading of Marsili plain (Figs 4 and 5). Overall, the spreading rate varied in the course of time. The start of oceanization of Vavilov and Marsili basins has been related to hyperextension and low-standing volcanism exhibiting round-shaped magnetic anomaly. Between ~5 and 1.87 Ma and between ~1 Ma and the Recent, conjugate margins volcanism (see Supplementary Info S2) has been accompanied by bathyal seamount volcanism linked to minor extension. Thus, it appears that oceanic accretion saw alternating intervals dominated by horizontal or vertical tectonic deformation associated to eruption of low- or high-standing volcanoes, respectively.

### Peri-bathyal magmas that are coeval with oceanic opening between 20 and 16 Ma

#### European lithosphere

Volcanic rocks of continental margin showing composition from basalt and andesite to ryolite, and K/Ar datings between ~19.8 and 15.8 Ma (~Burdigalian; Fig. 2B) are found in the graben of western Sardinia, in southern Corsica and Mallorca island (European lithosphere). Literature geochemistry and geochronology data of the calc-alkaline rocks which erupted concomitantly with Sardinia-Provence seafloor spreading are listed in the online Supplementary Dataset S4. The conjugate margin volcanism which preceded seafloor spreading between Provence (SE France) and Corsica-Sardinia is described by Supplementary Info S2. Moreover, Supplementary Info S3 considers the alkaline (anorogenic) basalt volcanism. At site 25 (Ligurian Sea, Fig. 2B) the presence of alkaline basalt volcanism has been recognized by French authors^16^ (see also Rollet *et al.*, 2002 at *Info S2*).

### Mediterranean orogen of Alpine age

In the Betic coastal area (southern Spain) crop out magmatics with basic to acidic composition^24^,^33^,^31^. The Malaga-Marbella dikes (site 14, western Betics) form W-E trending westwards translated bodies. The rootless dike swarm composition, tholeiitic and transitional to calc-alkaline, ranges from low-K basaltic andesite to medium-K andesite (samples AM24 to FG22; Supplementary Dataset S4). The basaltic dikes yielded ^40^Ar/^39^Ar ages of 17.4, 17.7, 19.8, 17.4; however 30.2 and 33.6 Ma are reported, too^33^,^34^. Granite dike from the Malaga area (site 14; sample MI22), granite clast from ODP Hole 977 (site 15, sample 7646), diorite clast from Carboneras (site 10; eastern Betics) and dacite from Mar Menor (site 17; sample MM2703) yielded ^40^Ar/^39^Ar ages of 18.5, 17.6, 18.9 and 18.5 Ma, respectively^31^,^32^,^33^. Based on available evidence, only the age values of tholeiitic rocks between ~19.8 and 17.4 Ma appear to be geologically meaningful since they are comparable with the values of the acidic high-K-rocks - generally better datable than the low-K ones with K/Ar and Ar/Ar know-how. Intrusive rocks from the Sierra Cabrera (site 16) yielded Rb/Sr dates of 20.4 and 18.8 Ma^34^.

Figure 2B shows that intrusives and volcanics are widespread in the Algerian margin^35^,^36^. Granodiorites are exposed in Thenia (site 10a; sample T22; Supplementary Dataset S4), monzonites and diorites in Bejaia-Amizour (site n. 11; samples A12, A1, A9). Cordierite-granites (samples U3, L61), gabbros and rhyolites (sample C1) crop out in the vulcano-plutonic complex of Cap Bougaroun-Collo (20 × 10 km; site 12), rhyolites in El Milia (about 30 km to the south of site 12); and tholeiitic basalts in Dellys (site 19). The space-time distribution of magmatic rocks that are not coeval with oceanic opening of the late Miocene and late Pliocene is considered in Supplementary Info S2.

### Relationships between oceanization and peri-bathyal magmas

#### Age distribution and start of WNW-directed subduction

An age histogram illustrates the geochronology data of magmatics from the Provence-Corsica-Sardinia-Tyrrhenian region and the peninsular Italy since the Oligocene (Fig. 4a). Figures 4b and 5 show that late-Miocene, along-strike magmatism consists of basalts and granitoids. These igneous rocks erupted respectively in the Vavilov plain (oceanic spreading OS2) and on the thinned continental crust of the north Tyrrhenian^37^. In the Burdigalian, seafloor spreading of the Sardinia-Provence basin (OS1) has been about concomitant with peak volcanism that accompanied the rotation of the Corsica-Sardinian block. The oceanization of the western part of OAA, too, has been accompanied by eruptive activity along the Algerian and Betic-Balearic conjugate continental margins (Fig. 2). On the other side, oceanic spreading OS2 and OS3 formed the Vavilov and Marsili plains (Fig. 4) while conjugate margin volcanism was absent or scarce.

**Figure 6:**
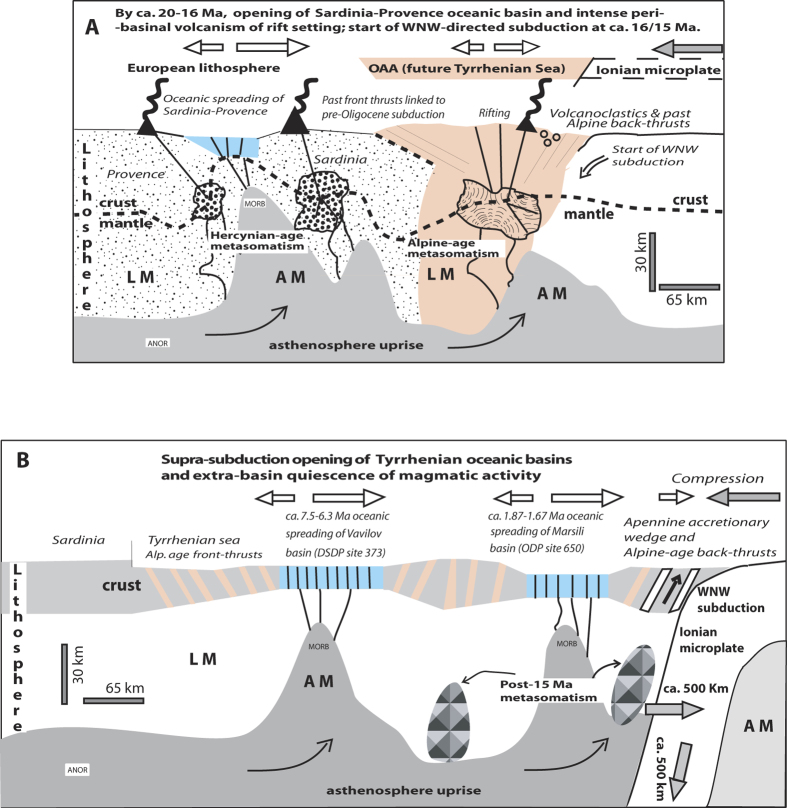
Schematic lithosphere-scale cross sections: (A), from Provence (France) to the Ionian area (location in Fig. 3B); the oceanic spreading of Burdigalian age (between ~20 and 16 Ma) produced the Sardinia-Provencal basin (Sa-Pr; European lithosphere); (B), from Sardinia to the Vavilov - Marsili oceanic basins and the Ionian (location in Fig. 5). AM = asthenospheric mantle, LM = lithospheric mantle, MORB = Mid-Ocean-Ridge-Basalt magma source in AM, ANOR = Anorogenic (alkaline, ocean-island-basalt) magma source in AM. It has here been assumed that the past lithosphere thickness of the Alpine-Betic orogen was comparable to that of nowadays Alps. Emplacement of the calc-alkaline magmas before start of WNW-directed subduction implies that the metasomatic modification of the corresponding igneous sources has been produced by lithosphere shortening of Hercynian or Alpine age. Because the orogenic accretion processes are repeated in time, Hercynian remnants are likely present in the metasomatized bodies of Alpine-age, and Alpine remnants in the bodies of Apennine-age. The figure was created by the author.

Such space-time distribution indicates that the Burdigalian-age oceanization on one side, and those of the late Miocene and late Pliocene on the other, are probably linked to clearly distinct tectonic environment. This reconstruction considers that Burdigalian seafloor spreading, and Oligocene-Aquitanian rifting, and calc-alkaline volcanism developed in the absence of subduction (Fig. 6A). The increase in volcanogenic allochthonous rocks showing Burdigalian age is reported in Supplementary info S3. The waning of allochthonous volcanoclastic deposits at ~16/15 Ma (Burdigalian-Langhian boundary^28^; Figs 2B and 6B) would accompany the nascence of WNW-ditected subduction of African plate under the eastern segment of OAA retro-belt (future Tyrrhenian Sea; Supplementary info S3). The novel lithosphere plunge of reversed polarity would go with the eruption of comendites (SW Sardinia, European plate), and lamproites (NE Corsica, eastern part of OAA; Supplementary Dataset S4).

## Discussion

### Mature and failed rift

Overall, rift tectonism is either of “failed” (aborted) or “mature” type: the former is linked to continental thinning typically characterized by horst-graben formation, and the latter reaches the eventual stage of oceanization. In various regions of the West Mediterranean and surroundings (e.g., the Valencia basin, the Alboran Sea, the Sicily Channel, the Aegean Sea, the Rheintal Valley, the Rhone Valley, Limagne-Massif-Central-Bresse) Tertiary-Quaternary rifting of “failed” type produced only continental thinning and stretching. “Mature” rift can be distinguished from aborted rift spatially or temporally. A spatial distinction is found in the Tyrrhenian Sea as late Miocene rift activity of “failed” and “mature” type is found respectively in its northern and southern parts. Modest extension (incomplete rift)^37^ accompanied the magmatism of granitoid nature of the north Tyrrhenian seafloor (Fig. 5). On the other hand, strong extensional deformation accompanied the Vavilov plain volcanism of MORB type to the south^29^. Granitoid magmas are widespread in the north Tyrrhenian, whereas basalt volcanism appears limited to the eastern rim of the Vavilov plain (DSDP well-373A; Fig. 3). Spatial distinction is also manifest in the Mediterranean OAA during the Burdigalian age. In fact, rifting of “failed” and “mature” type, in the absence of subduction, occur respectively to the east (future Tyrrhenian sea) and the west of the CTFZ (north Algerian basin). Further in the past, the Permo-Triassic “failed” rift of the southern Alps could have been associated with the distant opening of the Permo-Triassic Tethys. Regarding temporal distinction, rift/spreading transition of the Mediterranean OAA shows Early-Miocene, Late-Miocene and Late-Pliocene ages.

#### Nascence of WNW-directed subduction and the fate of volcanoclastic rocks

Pre-Oligocene (>33/32 Ma) lithosphere thickening above the SE-subduction of the European lithosphere produced the fore-belt and retro-belt of the Mediterranean-Tyrrhenian OAA^3^,^6^. Various authors and this reconstruction, consider that the alpine-age retro-belt is present in the internal part of the Apennines, possessing similar vergence to that of the future external part^1^,^6^ thus facilitating commencement of WNW-directed subduction of the African plate. Overall, intra-mountain lithosphere rupture and volcanism of calc-alkaline nature, produce fault-bounded horst, exposing crystalline-metamorphic and volcanic rocks, which alternate with grabens that contain thick deposits of volcanoclastic and siliciclastic nature^8^,^9^,^38^. The Supplementary Info S3, and Dataset S5, S6 describe the calc-alkaline volcanoclastic layers of Oligocene – Burdigalian age, which are found as allochthonous bodies in the Apennines in the absence of the emission centres. The enigmatic locality of the lost centres (Sardinia, Tyrrhenian Sea, Adriatic foreland) has been discussed by various authors (Supplementary Info S3). This reconstruction tentatively contributes to the discussion, considering that, at the nascence of the WNW subduction and of Apennine thrusting, the proposed upper plate source area of the allochthonous volcanoclastic rocks was probably affected by inversion tectonics, in which a compressional stage follows the extension-dominated stage. The fate of volcanoclastics might have been determined by the significant change of the tectonic mode affecting their sites of origin in the Tyrrhenian OAA. By the beginning of the WNW subduction, inversion tectonics would induce the initial down faulting of the original horst volcanoes (past topographic highs) and the upthrust of fault-bounded grabens bearing volcanoclastic deposits (former lows). In the more orthodox concept of West-Mediterranean-Tyrrhenian evolution, the persistent WNW subduction of the last 33/32 Ma, would exclude horst-graben inversion tectonics. The presence of rifting and calc-alkaline volcanism in the lack of ongoing subduction most probably implies that igneous sources have been metasomatized by crustal material brought downwards during previous period(s) of lithosphere shortening^11^,^12^,^13^,^14^.

#### The Oligocene of the Western Alps

The study area may have had important geological and temporal connections with the Oligocene Alps. The compression tectonics of the Western Alps has been supplanted by extension and lithosphere thinning that lasted from the start of the Oligocene until the late-early Miocene (~20 Ma)^11^,^39^,^40^. Rifting and increase in geothermal gradient produced HT/LP metamorphism, which was accompanied by generation and eruption of calc-alkaline melts between the Oligocene and early Miocene^40^. By the end of early Miocene, igneous activity and extension of the Alps ceased and the orogenic accretion resumed. The resumption of lithosphere shortening of the Alps appears to be temporally related to the onset of oceanic opening of the West Mediterranean. The reversal of subduction polarity and its geotectonic implications have been studied at the junction between the Western Alps and the Northern Apennines^4^. The authors recognize that polarity inversion started at the Eocene/Oligocene boundary, concomitantly with Alps’s extensional setting and Apennines’s thrusting. However, the authors consider also that subduction of true Apennine type has taken place only beginning from the late Miocene accompanied by the Calabrian slab pull. In view of this, a long-lived process of subduction flipping could have taken about 20 Ma.

#### Tectonics and magmatism

After the pre-Oligocene alpine accretion linked to subduction with SE polarity, the tectonic and magmatic activity of Oligocene-Burdigalian age can be distiguished from that of the Langhian-Recent. In the absence of subduction, rifting and magmatism showing acidic calc-alkaline nature and Oligocene-Aquitanian age (~33/32–20 Ma) were followed by sea-floor spreading (~20–16 Ma) and coeval calc-alkaline volcanism with basic to acidic composition around the oceanic domain. The start of WNW-directed subduction by the Burdigalian/Langhian transition preceded seafloor spreading of the Vavilov plain. Supra-subduction extension and basalt volcanism of Tyrrhenian seafloor have been discontinuous. Strong extension and scarce low-standing volcanism alternated with seamount volcanism linked to weak extension (Supplementary Info S2). MORB-type lavas (ODP well-655) created the modest elevation of 4.3 Ma old Gortani ridge (Fig. 3), located NW from the low-standing volcano of DSDP well-373. Afterwards, MORB volcanism migrated only towards the hinge zone. In the course of ESE-directed migration, from Gortani ridge to the axial volcanoes of Vavilov (<2.6/2.4 Ma; pre-Olduvai Matuyama^26^) and Marsili (<0.8 Ma; the Brunhes chron), the seamount elevation gradually increased. Eventually, large magma input formed the over-fed Marsili volcano, the last of the “sui generis” spreading axes of Tyrrhenian seafloor^25^. By the final stage of Vavilov plain oceanization (about <0.5 Ma), weak horizontal deformation went with eruption of alkaline basalt flows on the summit of Vavilov volcano (Fig. 5, and Supplementary Info S2).

## Conclusions

Pre-Oligocene, SE-directed flat subduction of the European plate produced the submerged orogen of the West-Mediterranean-Tyrrhenian region. Subsequently, WNW-directed steep subduction of the African plate accompanied oceanization of the Tyrrhenian basin, the segment of the submerged orogen to the east of CTFZ. Post-orogenic continental extension and calc-alkaline volcanism initiated in the Oligocene, in the Burdigalian (~20–16 Ma) reached the stage of an oceanic opening in the European plate (Sardinia-Provence basin) and the western segment of the submerged orogen (north Algeria basin). In this same time period, volcanism of calc-alkaline type was widespread on the margins of the oceanic domain. By contrast, the oceanic plains of late-Miocene Vavilov and late-Pliocene Marsili, originated when across-strike volcanism had been absent or scarce. The contrast between abundance and lack of conjugate margins’ volcanism, at various times of the seafloor opening, would turn out to be due to the diversity of the geotectonic setting. If so, from Early Oligocene to the Burdigalian/Langhian boundary continental extension seafloor spreading and calc-alkaline volcanism developed in rift setting, in the absence of subduction. The WNW-directed, steep subduction under the submerged and stretched orogen of alpine age, probably took place only in the last ~16/15 Ma (after the waning of Burdigalian sea-floor spreading). This reconstruction indicates that calc-alkaline volcanism is not linked exclusively to subduction. It appears that only the Tyrrhenian oceanization occurred in supra-subduction setting, after ~18/16 Ma between the conclusion of the SE-directed flat subduction and the nascence of steep WNW descent, representing the Alpine and the Apenninic mode of lithosphere consumption, respectively.

## Additional Information

**How to cite this article**: Savelli, C. Fast Episodes of West-Mediterranean-Tyrrhenian Oceanic Opening and Revisited Relations with Tectonic Setting. *Sci. Rep.* **5**, 14271; doi: 10.1038/Srep14271 (2015).

## Supplementary Material

Supplementary Information S1, S2, S3

Supplementary Information S4

Supplementary Information S6

Supplementary Information S5
