# Supplementary material for: Fast Episodes of West-Mediterranean-Tyrrhenian Oceanic Opening and Revisited Relations with Tectonic Setting
Source: Sci Rep. 2015 Sep 22;5:14271. doi: 10.1038/srep14271 (PMC4585736; doi:10.1038/srep14271)
Supplement: Supplementary Information S1, S2, S3 [file srep14271-s1.pdf]

FAST EPISODES OF WEST-MEDITERRANEAN-TYRRHENIAN OCEANIC OPENING AND REVISITED RELATIONS WITH TECTONIC SETTING

Carlo Savelli

Consiglio Nazionale delle Ricerche, Istituto di Scienze del Mare, via P. Gobetti, 101, 40129, Bologna, Italy
E-mail address: carlo.savelli@bo.ismar.cnr.it

<u>SUPPLEMENTARY MATERIAL</u>

Supplementary Info - S1: Brief description of previous tectonic reconstructions

It is generally considered that calc-alkaline volcanism, oceanic spreading and bending of the Maghrebid-Apennines belt were linked to roll-back of WNW-directed subduction of African plate from early Oligocene to Recent. For the most part, the authors call for the arc environment on the assumption that calc-alkaline magmas and oceanic opening probably require concomitant slab retreat extending from the north Apennines to western Maghreb (see, e.g., *Malinverno and Ryan*, 1986; *Lonergan and White*, 1997; *Carminati et al*., 1998a; *Wilson and Bianchini*, 1999; *Argnani and. Savelli,* 1999; *Maury et al*., 2000; *Cibin et al*., 2001; *Barruol and Granet*, 2002; *Rosenbaum et al*., 2002; *Spakman and Wortel*, 2004; *Martin*, 2006; *Scrocca et al*., 2005; *Heymes et al*., 2008; *Vignaroli et. al*., 2008)**.** In the subduction-controlled environment has been considered the occurrence of: slab break-off (*Carminati et al*., 1998a, b; *Benito et al*., 1999; *Hoernle et al*., 1999; *Maury et al*., 2000; *Cavazza et al*., 2004), slab tear (*Patacca et al*., 1990; *Marani and Trua*, 2002; *Faccenna et al*., 2004, 2007; *Chiarabba et al*., 2008; *Rosenbaum et al*., 2008), inversion of subduction polarity (see, e.g., *Boccaletti et al*., 1971; *Rehault et al*., 1984; *Doglioni et al*., 1998, 2004; *Gueguen et al*., 1998; *Peccerillo*, 1999; *Savelli,* 2002, 2011), slab roll-back without polarity inversion (*Beccaluva et al., 1994*; *Argnani et al*.,1995; *Lonergan and White*, 1997), and delamination of subducted lithosphere (*Serri et al*., 1993; *Hoernle et al.,* 1999). Among the extension-controlled processes, various authors consider convective thinning of lithospheric mantle and orogenic collapse (see, e.g., *Doglioni,* 1995*; Turner et al*., 1999; *Doblas et al*., 2007). *Doglioni* (1992) and *Keller et al.* (1994) propose that the eastward mantle flow is at the origin of back-arc extension and consecutive oceanic opening. *Doglioni et alii* (1999a, b) suggest that mantle flow results in the contrasting modes of the steep and flat subduction zones. The "two subductions" concept considers that flat E-subduction zones are associated to uplifting mountains (e.g., the western Alps) and steep W-subduction zones to small elevation of the chain (the Apennines).

Lavecchia and Stoppa (1990, 1996) and *Bell et alii (*2006) believe that east-dipping detachment faults of the Sardinia/Tyrrhenian region and volcanic activity associated with eruption of carbonatite rocks and deep-seated CO_2 emission of the Apennine region took place in response to the ascent of intra-continental plume. Diverse, but not mutually exclusive processes, occurred in the western passive and the eastern active margin (*Kastens et al.,* 1988; *Keller et al*., 1994; *Sartori et al*., 2004). While the passive continental Corsica-Sardinian margin is thinned and stretched by detachment faults, back-arc extension and arc volcanism migrate eastwards. This reconstruction might recall the idea of *Tatsumi et alii* (1990) that asthenosphere injection into the mantle wedge forced slab steepening and back-arc extension. Some authors (*Turner et al*., 1999; *Doblas et al*., 2007) consider that the post-collision Betic-Alboran magmatism was a consequence of convective removal of lithospheric mantle and lower crust in the absence of subduction. Local comenditic rocks from Sardinia and lamproitic rocks from Alpine Corsica are thought to reflect a tectonic environment of continental extension rather than subduction (*Morra et al*., 1994; *Prelevic et al*., 2007). *Mantovani et alii* (2002) and *Viti et alii* (2009) consider that Africa-Europe plate motions drove wedge extrusion and formation of the trench-arc-back-arc sequence.

The slab beneath the Gibraltar arc is regarded as the counterpart of the Calabrian arc at the eastern termination of the WNW subduction beneath the Maghrebid-Apennines belt (*Lonergan and White*, 1997; *Gutscher et al*., 2002; *Faccenna et al*., 2004; *Doblas et al*., 2007). In this view, the former arc migrated to the west and the latter to the east. It has been proposed that the Sardinia calc-alkaline magmas originate from igneous sources which were metasomatized during plate convergence of Hercynian age (*Rehault et al*., 1984; *Savelli*, 2002*,* 2005). Figure 2 shows that the pre-Oligocene (> 33.9 Ma; *International Stratigraphic Chart,* 2009) NW-verging front of shortening due to collision of the European and African

plates was accompanied by formation of SE-verging back-thrusts which will be the hinge zone of future WNW subduction of African lithosphere beneath the Maghrebid-Apennines belt. The similar SE-vergence of the inherited back-thrusts of Mediterranean OAA and the thrusts of the Apennines (*Keller et al*., 1994; *Doglioni*, 1998; *Gueguen et al*., 1998; *Scrocca* et al., 2005) causes difficulties in determining initiation of the WNW subduction. Some authors (*Carminati et al*., 1998b; *Cibin et al*., 2001; *Faccenna et al*., 2004; *Rosenbaum et al*., 2008; *Conticelli et al*., 2009) propose that subduction started in early Oligocene (ca. 34/30 Ma), and some others (*Gueguen et al*., 1997, 1998; *Guerrera et al*., 1993, 2004, 2005) in late Oligocene (ca. 25 - 23 Ma or late Oligocene-Aquitanian time, ca. 25 – 20 Ma). Subduction beneath the northern part of the Tyrrhenian/Apennine system initiated probably not earlier than ca. 20 – 14 Ma (early-middle Miocene; *Savelli* 2000; *Guerrera et al*., 2012). *Maury et alii* (2000) propose 16 Ma for slab break-off and commencement of calc-alkaline magmatism along the Maghreb margin. Given spreading rate of ca. 5 cm/a and slab depth of ca. 500 km beneath the Tyrrhenian basin, *Francalanci and Manetti* (1994) consider subduction duration of 10 Ma .

Supplementary Info - S2: Periods of weak extension and volcanism not coeval with the oceanic openings

Oligocene - Aquitanian (ca. 33/32 - 20 Ma)

- European Plate

East of the Catalan-Tunisian fracture zone, the island of Sardinia saw intense rift activity. In the west Sardinia graben (Fig. 3/A, main text), lava flows and domes - often intruded by dikes and autobrecciated - are accompanied by ignimbrites, pyroclastics and pillow basalts. A series of individual volcanic complexes is the typical product of Sardinia volcanism. Rock composition varies from basalt to andesite dacite rhyolite, the serial type from tholeiitic and medium-K calc-alkaline to high-K calc-alkaline/shoshonitic, and the age from ca. 29 to 12 Ma (*Deriu*, 1962; *Coulon et al*., 1974; *Coulon and Dupuy*, 1975; *Savelli et al*., 1979; *Dostal et al*., 1982; *Beccaluva et al*., 1994; *Argnani et al*., 1995; *Conte*, 1997; *Lustrino et al*., 2004; *Gattacceca et al*., 2007). Andesitic lavas and ignimbritic rhyolites are the prevailing lithotype of the island (Tab. 1). The Sarroch volcanic complex (site 5; Fig.3/A) is made of basalts, basaltic andesites and andesites (samples SH-32, SH-55, SH-48 of Tab.1; *Conte*, 1997). Basalts and andesites crop out in Cixerri (site n. 6; *Savelli et al*., 1979); microdiorites, rhyolitic ignombrites, dacites and andesites in Alghero-Oniferi (site 7; *Bellon*, 1981, *Gattacceca et al*., 2007); basalts, basaltic andesites and andesites in Bosano-Logudoro-Capo Marargiu (site 8). In Provence are present andesites, dacites and subvolcanic microdiorites (sites 3, 4). Andesite and dacite are lava flows (samples 77S4F, Est96-2, PR4), gabbro xenolith is present in dacite lava (sample PR7a from Esterel area), and andesite clasts (in the Villeneuve-Loubet conglomerate near Nice (sample PR2) (see note at foot of a previous page).

In Valencia basin, to the west of Catalan-Tunisian fracture zone offshore exploration wells sampled early Miocene sediments bearing pyroclastics of andesitic to rhyolitic composition (site 1; *Marti' et al*., 1992). The DSDP hole 123 drilled pyroclastics (site 2; samples n. 2/6a and 2/6b) of dacitic and rhyolitic composition which yielded K/Ar dates of 24.4, 21.9 and 20.8 Ma (*Ryan et al*., 1972).

- Mediterranean Orogen of Alpine age

West of the Catalan-Tunisian fracture zone, ca. 24.8 to 20.8 Ma old leuco-granite clasts are present in the Betic area (site 10; *Bellon et al*., 1983). Rhyolitic pyroclastics (22-21 Ma, site 9) and volcanogenic turbidites show Aquitanian-Burdigalian age (Algeciras, *site 2 - in italics*, Fig. 3). In the Algerian Maghreb, the granodiorite from Bejaja-Amizour (Great Kabylia) and the granitoid from Beni Toufout (Little Kabylia) show late Oligocene (24.4 Ma; site 11) and Aquitanian age (22 Ma; site 12), respectively (*Bellon*, 1981). These intrusions are slightly younger than the peak thermo-metamorphism of the Kabylia crystalline basement which is dated at ca. 25 Ma (*Monie' et al.,* 1995).

Langhian - Tortonian (ca. 16 - 7.5 Ma)

- European Plate

Ignimbritic rhyodacites and andesites (sites 42, 43), comendites (site 44), and andesites (near to s. 6) crop out in northern, southwestern and southern Sardinia respectively. Shoshonites are found in the Sardinian offshore (site 45; sample Sar1.03; *Mascle et al*., 2001) and andesites in the Ligurian offshore (*Rollet et al*., 2002; p. -17).

Mediterranean Orogen of Alpine age

Alboran region West of the Catalan-Tunisian fracture zone, magmas erupted mostly along lineation zones trending SW-NE (*Hernandez et al*., 1987; *Duggen et al*., 2004), W-E (*Torres Roldan et al*., 1986) and WSW-ENE (*Hoernle et al*., 1999). The SW-NE lineation (Trans-Alboran fracture zone) extends from the Trois Fourches promontory of Morocco to the Betic area of Cabo de Gata (sites 30, 26, 27). Along such lineation ODP Hole 977 (site 15) recovered gravel pebbles which have dacite, basalt (sample 7523) and rhyolite composition (sample 7521) and ages between 12.1 and 9.3 Ma (*Hoernle et al*., 1999; *Duggen et al*., 2004). Pebbles from Hole 978 (near to s. 15) exhibit composition from basalt to rhyolite and serial type ranging from tholeiite to shoshonite (*Hoernle et al*., 1999). The pebbles below early Pliocene sediment of ODP Holes 977 and 978 probably derived from the widespread late Miocene erosion process. At the Yusuf ridge is present andesite (10.7 Ma; sample CYA 5-6) and at Mansour seamount basaltic andesite (8.7 Ma: sample POS III-D-1; *Duggen et al*., 2004). Sea-floor rhyolites (9.4-9.3 Ma; site 26; sample CYA3-11) accompanied by subaerial tholeiitic lavas and andesitic pyroclastics are present along the lineation trending WSW-ENE of the submerged ridge near to the Alboran island (*Duggen et al*., 2004; *Gill et al*., 2004).

Betic region. Volcanic activity was intense in the Betic region between ca. 12 and 9 Ma. The 8.9, 8.8 Ma old basalt and latite of Mazarron (site 28) show high-K calc-alkaline and shoshonitic type, respectively (samples B302 and MAZ8; *Benito et al.*, 1999; *Turner et al.*, 1999). Granodiorites, diorites, rhyolites, dacites, andesites, basaltic andesites and basalts erupted in the time span from ca. 15 to 8-7 Ma in the Cabo de Gata region (site 27; *Bellon et al.*, 1983; *Di Battistini et al.*, 1987; *Hernandez et al.*, 1987; *Turner et al.*, 1999). The ca. 1000 m thick CA lavas of Cabo de Gata exhibit K/Ar age between 15.2 and 7.9 Ma (*Bellon et al.*, 1983). *Di Battistini et alii* (1987) report that 12.1 to 9.2 Ma old amphibole-bearing andesites dacites and rhyolites overlie pyroxene-bearing andesites (8.7 to 7.5 Ma; samples CG200 599-9, Sp317, Sp333, Sp253).

Morocco Rif. Pyroclastics and block lavas of basaltic-andesitic and andesitic, medium-K calc-alkaline composition from Ras Tarf (site 29; sample 116-72) yielded K/Ar dates of 15, 13.1, 12.1 Ma (*Bellon*, 1981; *El Azzouzi et al.*, 1999; *Maury et a*l., 2000). More to the east at the southern end of Transalboran shear zone, rhyolites crop out in the Trois Fourches promontory (9.8 Ma; site 30; sample G44; *El Bakkali et al.*, 1998). Rocks of high-K calc-alkaline, granodioritic and andesitic composition (samples G8, G1, G15) from the adjacent volcano/plutonic complex of Gourougou show ages of 8.1, 7.9, 7.7 Ma respectively (*El Bakkali et al.*, 1998; *El Azouzi et al.*, 1999). In addition, the Gourougou area is characterized by eruption of shoshonitic rocks showing composition from basalt to trachyte (samples G47, G74) and K/Ar age older than the Tortonian (between ca. 7.0 and 5.4 Ma).

Algerian Maghreb. In the Great Kabilya region of Algeria (site 20) the pre-Langhian magmatics were followed by emplacement of latite dikes with age of 12.4 Ma. *Louni Hacini et al.* (1995) report K/Ar age of 15 Ma for the rhyolitic dome of Sahel of Oran (site 31) which was followed by high-K calc-alkaline andesites and dacites with K/Ar ages between 11.7 and 7.2 Ma (dike sample OR10), and 7.5 Ma old latites (site 32; latite dome of M' Sirda; sample ORbd1). Rhyolitic dikes cutting the Thenia granodiorite of Burdigalian age (site 10a; sample T 36) have been dated 14-12 Ma. *Bellon* (1981) indicates 16-15 Ma for the rhyolites and rhyodacites from Menacer area (site 36), *Maury et alii* (2000) 13-11 Ma for K-rich granites from the Cherchel area (site 34) and *Belanteur et alii.* (1995) 11.8 Ma for basaltic andesite from the area of Dellys (near to site 19; sample DLBO). According to *Bellon* (1981) andesites and shoshonites of the Cherchel area (site 35) gave K/Ar ages of 13.1/12.4 and 9.0 Ma (respectively), and andesites of Kef Hahouner (site 39) 10.9/9.3 Ma. Ca. 15.3 Ma cordierite-bearing granitoids of Djebel Filfill (Little Kabylia; site n. 37; sample II-6) are of K-rich type. Granite and diorite crop out in Annaba (K/Ar age of 15.9 and 15.8 Ma; site 38, near to the western margin of Little Kabylia; *Bellon*, 1981); gabbros in Cap de Fer-Annaba (sample VPE-271) and basalts in Cap Djinet (13.7 and 12.2 Ma; site 36; samples Dj2, Dj1; *Fourcade et al.*, 2001).

Tunisia. The ca. 13 Ma old cordierite-bearing granitoid intrusion of Oued Belif (site 40; Nefza region) was followed by eruption of dacites and rhyodacites between ca. 12.9 - 8.2 Ma; cordierite-bearing, K-rich granitoids and rhyolites crop out in the island of La Galite (ca. 14.2 and 10 Ma; site n. 41; *Bellon*, 1981; *Maury et al.*, 2000).

NE Corsica. East of the Catalan-Tunisian fracture zone, at Sisco crops out a dike body showing lamproitic composition and K/Ar age of ca. 15.0 Ma (site n. 46; *Civetta et al.*, 1978; *Savelli*, 2000; *Conticelli et al.*, 2007, 2009). This rock has high contents of compatible (K, Rb, Th, Sr, Ba), incompatible (Mg, Cr, Ni) and light rare earth elements, as well as high Sr (0.71228) and low Nd (0.51215/6) initial isotopic ratios (Tab. 1).

Pliocene (ca. 5.4 - 1.8 Ma; Tyrrhenian area)

Major sources of detailed documentation for the Pliocene and Quaternary magmatic episodes in and around the Tyrrhenian sea (Figs. 5, 6 *and supplementary Fig. S1*) are found in *Selli et alii* (1977), *Savelli* (1984, 2002), *Serri et alii* (1993), *Faggioni et alii* (1995), *Argnani and Savelli*, (1999, 2001), *Peccerillo* (1999), *Sartori et alii* (2004). After the calc-alkaline volcanic cycle of Oligocene - Langhian age and ca. 7 Ma of magmatic quiescence, Sardinia was affected by basaltic volcanism showing alkaline nature and age from ca. 5.4 to 1.8 Ma (*Beccaluva et al.*, 1977; *Savelli*, 2002; *Lustrino et al.*, 2004; and references therein). Pliocene basalt volcanism is widespread in the Tyrrhenian seafloor. Alkaline basalts (3.0-2.7 Ma) occur in Magnaghi seamount, MORB-type lavas in the Gortani ridge (4.1 Ma; ODP site 655) and calc-alkaline basalts (2.6 Ma) above ultramafic rock of ODP site 651A. The age of Vavilov volcano is < 2.4 Ma if its magnetic anomaly belongs to the Matuyama chron. Figure 7 (main text) shows that Pliocene magmatics are present on the west of the

Tuscan-Roman-Campanian area of abundant Quaternary volcanism. In the Aceste and Anchise seamounts are found volcanics respectively of alkaline and calc-alkaline type.

Quaternary (< 1.2 Ma – Recent; Tyrrhenian area)

Calc-alkaline basalt volcanism (< 0.8 Ma; *Selli et al*., 1977; *Savelli and Gasparotto*, 1994; *Faggioni et al*., 1995; *Marani and Trua*, 2002) and high angle faulting produce vertical accretion of the large axial seamount Marsili as fast subsidence affects the seamount itself and the contour bathyal plain. Such Quaternary vertical tectonic mode replaces the low-angle detachment faulting of fast horizontal spreading and basalt eruption at ODP site 650 of the late Pliocene. *Cocchi et al*. (2009) point out that Marsili basin evolves from pure lateral spreading to a superinflated seamount. In the part of the basin to the east of ODP site 650, the full spreading rate decreases from 3.1 cm/a since the post Olduvai part of the Matuyama (< 1.67 Ma) to 1.8 cm/a since the Brunhes chron (< 0.8 Ma) and the start of the vertical growth of the 3000 m high seafloor volcano.

In the Marsili basin, if the ca. 2 Ma age of opening for 110 km in WNW-ESE direction is true the full spreading rate was ca. 5.8 cm/a. *Nicolosi et al*. (2006), indicate spreading rate of 19 cm/a for the short-lived Olduvai chron. As the Marsili spreading should be restricted due to nearness of the continental crust, the large above-mentioned rate can be an overestimation. The MORB-like basalt injection at ODP site 650 represents initial oceanic spreading of localized punctiform nature in ambient shallow-water. Such short-lived magmatic emplacement can accompany strong tectonic extension. To the west of the ODP site 650, strong tectonic spreading could affect - hypothetically - the amagmatic thinned continental lithosphere of the bathymetric saddle which separates the bathyal plain of Vavilov from that of Marsili. In the Marsili basin as in the older Vavilov, lateral oceanic accretion evolves to vertical growth producing basaltic seamounts to the detriment of the opening rate. The landlocked Marsili volcano grew up in a more eastward position with respect to the early localized shallow-water spreading of the site ODP 650, whereas the axial volcanoes of the Vavilov plain develop in a more westward position with respect to the MORB-type basalts of the site DSDP 373A. In the Marsili basin, the transition from pure horizontal magma-poor spreading to strong volcanism and vertical accretion of Marsili seamount can be cause or effect, or both cause and effect, of strong slab instability. This geochronological recostruction of the Tyrrhenian opening might show that the Gortani ridge and the seamounts of Vavilov and Marsili can represent the evolution from pure lateral to vertical oceanic accretion with formation of short "sui generis" spreading axis.

During the last 1 Ma, abundant calc-alkaline volcanism affected the Tyrrhenian margin from the Aeolian islands and Vesuvius to the "Roman Province" and southern Tuscany (Main text Fig. 7). The figure shows also that alkaline-olivine basalts are present in the top of Vavilov seamount showing < 0.5 Ma (*Selli et al*., 1977; *Robin et al*., 1987; *Faggioni et al*., 1995), and in NW Sardinia (between ca. 0.8 and 0.2 Ma; *Savelli*, 1984, 2002). Alkaline contamination of Quaternary peri-Tyrrhenian calc-alkaline volcanics is described by various authors (see, e.g., *Ellam et al*., 1989; *Serri et al*., 1993; *Francalanci and Manetti*, 1994; *Argnani and Savelli*, 1999, 2001; *Peccerillo*, 1999; *Gasperini et al*. 2002; *Cadoux et al*., 2005; *De Astis et al*., 2006; *Rosenbaum et al*., 2008; *Trua et al*., 2004, 2010).

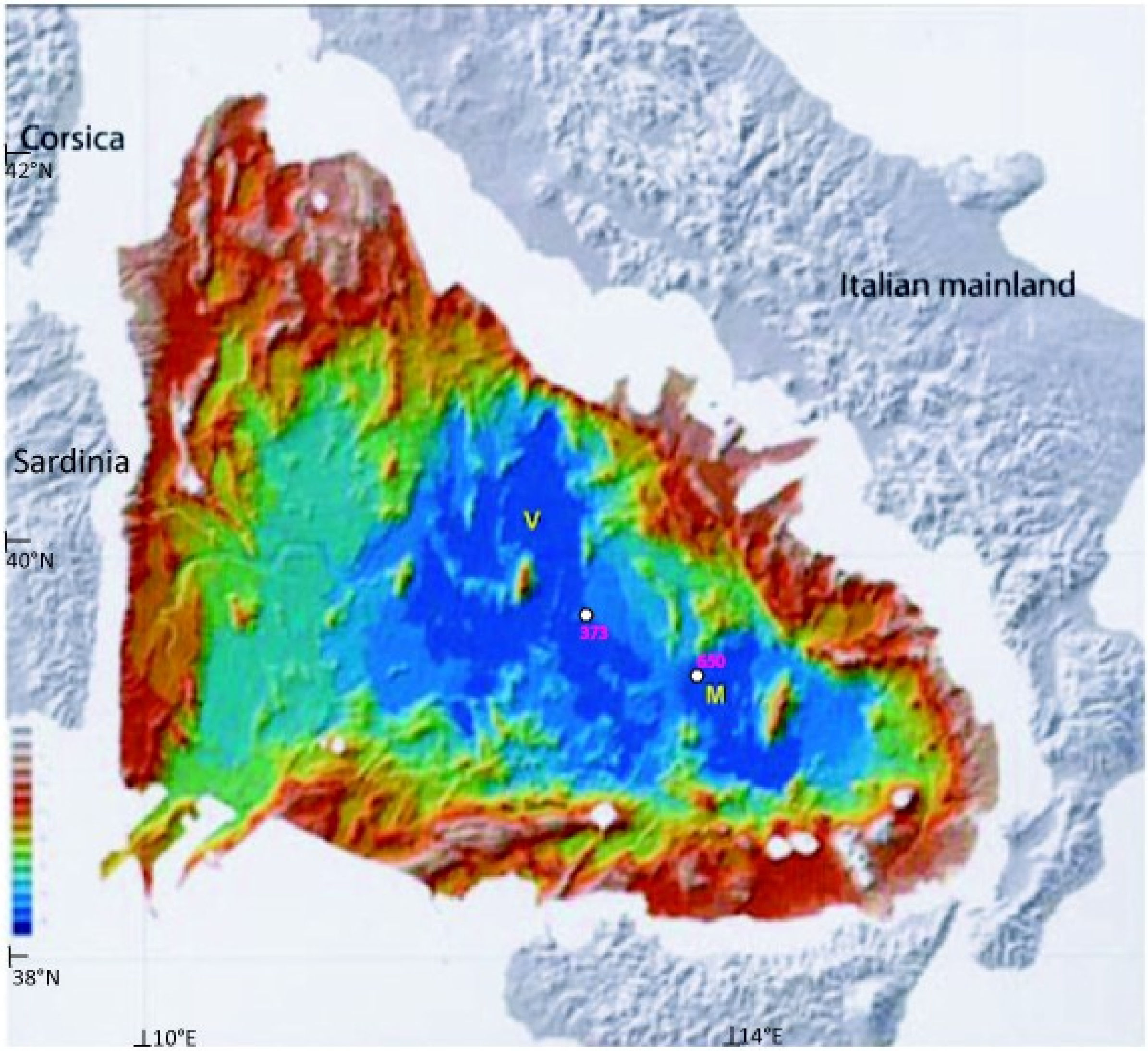

Figure S1. Shaded relief image of the Tyrrhenian seafloor below 200 m waterdepth. The Tyrrhenian bathyal plains of Vavilov (**V**) and Marsili (**M**) reach depth of 3600 m (dark blue). Their deep-seated basaltic crust has been drilled by DSDP well-373 and ODP well-650. From the "Memorie Descrittive della Carta Geologica d'Italia, vol. 64 (2004) ISPRA"; reproduced with permission of ISPRA.
http://www.isprambiente.gov.it/en/publications/technical-periodicals/descriptive-memories-of-the-geological-map-of/from-seafloor-to-deep-mantle-architecture-of-the

Supplementary Info – S3: Volcanogenic allochthons of the Apennines and anorogenic volcanism

Volcanoclastic sediments

The pervasive presence of allochthonous volcanoclastics in the absence of the corresponding emission bodies has attracted the attention of various authors (*Mezzetti et al*., 1964; *Riviere et al*.,1977; *Borsetti et al*., 1984; *Critelli et al*., 1990; *Guerrera et al*., 1993, 1998, 2005; *Amorosi et al*., 1994, 1995; *Campos Venuti et al*., 1997; *Cibin et al*., 1998, 2001; *Mattioli et al*., 2002). The rocks show age from the Oligocene to Burdigalian (Langhian), and calc-alkaline nature (Table 2). In Table 3 are listed the literature geochemistry data of the samples cited in this chapter and Table 2. The dilemma whether the source area was originally located in the Tyrrhenian OAA, to the east of the Catalan-Tunisian fracture zone, is object of debate. The provenance area and the tectonics causing disappearance of the primary emission bodies (the "lost" volcanoes) preservation of the volcanoclastics are related issues which are going to be considered more below. On the whole, volcanogenic sediments from the Apennines were associated to erosion, reworking and basinal slumping (mass-flow) of pyroclastic material and lava and mineral grains. Volcanoclastic sandstones with abundant glass and mineral fragments, tuffaceous and volcanolithic sandstones reflect sedimentation by turbidity currents and mixing with the terrigenous derive mostly from erosion of

crystalline-metamorphic basement. Fine-grained ash layers of pyroclastic fall which are often altered to clay minerals are also present. Some authors proposed the volcanoclastics derive from the coeval volcanism of western Sardinia (Tab. 3). Yet, due to its remote location the island is not a likely site of origin; the turbidite and gravity flow characteristics, coarse grain size (from sand to gravel and pebble) and thickness of volcanogenic sediments indicate a nearness of eruption and deposition sites (*Critelli et al.*, 1990; *Campos-Venuti et al.*, 1997; *De Capoa et al.*, 2002).

Early Oligocene.

Early Oligocene volcanoclastic beds are widespread in the northern Apennines (*Tateo*, 1993; *Cibin et al.*, 1998, 2001; *Mattioli et al.*, 2002). The Ranzano formation (l.s., sites *23, 24, 25;* Fig. 3, main text) is made up mainly of sands containing plagioclase grains of andesine-labradorite composition associated with glass shards and grains of amphibole, pyroxene, biotite and opaques which indicate primary composition of dacitic-andesitic type (samples 34D, 94, 128 of Tab. 2; *Cibin et al.*, 1998). The detritus is present in "pure" beds which contain around 70-80 % volcanic material and "mixed" beds where volcanic and non-volcanic grains are present in variable amount. Pebble-sized clasts from the Aveto-Petrignacola formation (sites *23, 24*) yielded amphibole Ar/Ar ages of 29.8-29 Ma which match the early Oligocene biostratigraphic evidence (*Mattioli et al.*, 2002). Clast composition varies from basalt and basaltic andesite to andesite, dacite and rhyolite (twelve samples; from MM2 to MM114). The lavas exhibit serial type from low- to medium- and high-K calc-alkaline, and the rhyolitic ignimbrites shoshonitic type. The volcanic sand beds of Reitano-Tusa and Stilo-Capo d'Orlando turbiditic successions (Calabro-Peloritani arc, sites n. *13*, *14*) show rhyolitic K-alkaline nature and early Oligocene age (*Faugeres et al.*, 1992; *Balogh et al.*, 2001; *Baruffini et al.*, 2002).

Oligocene - early Miocene.

Volcanogenic sands showing rhyolitic-rhyodacitic composition are found in the southern Apennines (sites *15, 16, 17; Critelli et al.*, 1990; *Guerrera et al.*, 1998, 2005; *De Capoa et al.,* 2002). Volcanic ash layers of the northern Apennines (Contignaco, Mongardino and Calderino; sites n. *25-26*) are associated with early Miocene clays (*Mezzetti et al.*, 1964; *Cibin et al.*, 2001). Sometimes, the volcanic ashes were modified and altered to various extent: the alteration degree was studied by *Mezzetti et al*ii (1964). The geochemistry diversity between separated shards and whole rock indicates the alteration of Calderino pyroclastics (sample 4). The alteration produced decrease of silica and alkalies, and CaO increase. Conversely, the Mongardino glass (sample 5) was only slightly altered into clay material. In volcanic arenites of the early Miocene San Mauro/Pollica formation (southern Apennines; *Crisci et al.*, 1988; *Critelli and Le Pera*, 1990) are present lava clasts of rhyodacitic - rhyolitic composition (sites *19-20*; samples S2, S3). The arenites contain fragments of plagioclase, quartz, sanidine and biotite; fresh plagioclase with anorthite content of 15 – 40 % shows embayed rims of allotriomorphic quartz.

Late Aquitanian – Burdigalian (Langhian)

Figure 3 shows that volcanogenic deposits of Late Aquitanian – Burdigalian age are scarce in Rif and Betics (*Maate et al.*, 1995; *Guerrera et al.*, 1998) and abundant in the Apennines. Turbidite sediments bearing fragments of rhyodacitic-andesitic rocks crop out in the Betic region (sites *1, 3 – in italics*; *Guerrera* et al. 1998, 2005). *Maate et al.* (1995) report fragments of basalt and andesite lava in turbidites from western Maghreb (sites *4, 5, 6*). The "Bisciaro" formation volcanoclastics of the north Apennine Umbria-Marche region (sites *27, 29*) yielded K/Ar ages between ca. 22.3 and 17.1 Ma (*Balogh et al.*, 1993). Rhyolitic-rhyodacitic glass shards and plagioclase phenocrysts are present in the "Bisciaro" type deposits of Montebello and Fossombrone (samples 3, B6/1, B8/7; B6(2), B8(1), 80, 80 mc; *Guerrera et al.*, 1986; *Coccioni et al.*, 1988). *Delle Rose et al.* (1994a, b) reported the occurrence of rhyolitic glass of shoshonitic type among the volcanoclastic layers of the Vicchio marls (site *29*). Vicchio glass shards are accompanied by altered andesite clasts and sand-sized grains of fresh andesine, plagioclase, biotite. Conglomerate beds with pebble-size lava clasts of rhyolitic - rhyodacitic composition (samples A II, C 13A, B 12), and volcanic arenites are present in the Gorgoglione flysch (site *22*; *Le Pera and Ventura*, 1994). The clasts contain phenocrysts of plagioclase (An 5-30), K-feldspar, quartz and biotite. *Langhian*. Pyroclastics of Langhian biostratigraphic age which bear rhyolitic glass shards and biotite crystals (site *11*; samples 7, 14) crop out in central Sicily (*Spadea and Carmisciano*; 1974).

The buried volcano of Pieve S. Stefano

In the northern Apennines (site 13a) the oil drilling of Pieve S. Stefano recovered calc-alkaline volcanics (*ENI/AGIP report - San Donato Milanese, June* 1984; *Anelli et al*., 1994). The 850 m thick igneous sequence ends at drilling depth of ca. 4900 m. The core samples m 3870-1 and -2 consist respectively of pyroxene-bearing andesite lava and andesite pyroclastics. The presence of "brown-red"colour surfaces likely suggests not less than ten eruptive events. The volcanic sequence was interrupted by two large anhydrite slivers belonging to the Triassic Burano formation. The evaporitic layers, up to one hundred meter thick were probably crucial for the strong tectonic displacement of this igneous sequence. Pervasive alteration produced the replacement of primary mineralogy with hydrothermal minerals. The increase with depth of epidote content and other hydrothermal alteration products of the deep-seated Pieve S. Stefano volcano supports the idea that the original eruptive sequence might have been preserved. Three samples of decalcified andesite grains from core interval 3864 – 3872 m yield radiogenic ^{40}Ar contents of (1.333, 1.179, 1.012) x 10^{-3} mm^3/gram. Apparent ages of 33.8, 30.0, 25.7 Ma are obtained by K_2O content of 1.21 %. Precisely repeated datings are not to be expected from the altered rocks of Pieve S. Stefano. If the average date of 29.8 Ma is a true date, it indicates the approximate timing of alteration rather than that of eruption.

Start of WNW-directed subduction and the "lost volcanoes".

Overall, postorogenic lithosphere rupture and volcanism of calc-alkaline nature produce alternance of fault-bounded horst, exposing crystalline-metamorphic and volcanic rocks, and grabens that contain thick deposits of volcanoclastic and siliciclastic nature (*Stewart*, 1978; *Hawkesworth et al*., 1995; *Hooper et al*., 1995). Calc-alkaline volcanoclastic deposits of Oligocene – Burdigalian age are found as allochthonous bodies in the Apennines in the absence of volcanic emission centres (Fig. 3). This geotectonic reconstruction considers that, at the nascence of WNW subduction and thrusting beneath the Apennines (ca. 16/15 Ma), the submerged Tyrrhenian OAA, source area of the allochthonous volcanoclastics was probably affected by commencement of inversion tectonics in which compression follows the extension-dominated phase. The fate of the volcanoclastics could have been determined by a significant change of tectonic mode in their former sites of origin. By the start of WNW subduction, inversion tectonics could initiate the down-faulting of the original horst volcanoes (past topographic highs) and the thrusting of the fault-bounded grabens bearing volcanoclastic deposits (former lows). Rupture of the original upper plate horst-graben architecture probably initiated gradual downdrag and upthrust motions which were at the origin of the loss of the original topographic highs (volcanic horsts) and preservation of the lows (fault-bounded grabens bearing volcanoclastic deposits). In the more orthodox concept of West-Mediterranean-Tyrrhenian evolution, the persistent WNW subduction of the last 33/32 Ma excludes horst-graben inversion in which a contractional phase follows the extensional phase.

Anorogenic (alkaline) volcanism

Localized anorogenic volcanics are associated to the extensive calc-alkaline (orogenic) magmatism of the Mediterranean-Tyrrhenian area. The anorogenics (mainly basalts of Na-alkaline composition), despite the minor amount, provide important constraints for tectonic reconstructions. Generally, the Mediterranean-Tyrrhenian anorogenic volcanism with time propagated supplanting the orogenic manifestations (see e.g., *Savelli*, 1988, 2002; *Marti' et al*., 1992; *Serri et al*., 1993; *Beccaluva et al*., 1994; *Marani and Trua*, 2002; *Cavazza et al*., 2004; *Faccenna et al*., 2007). The shoshonitic series of Morocco Rif was followed in time by scattered Na-alkaline basalts with K/Ar age between 5.6 and 1.5 Ma (*El Bakkali et al*., 1998).

In the Oran area, basalts of anorogenic - transitional serial character (site 33; sample OR13; Tab. 3) yielded K/Ar age of 8.8 Ma that overlaps temporally the orogenic products (*Louni Hacini et al*., 1995; *Coulon et al*., 2002). These authors report that Oran anorogenic volcanics erupted between ca. 4.0 and 0.8 Ma. A gradual transition from orogenic to intermediate and anorogenic serial affinity has been recognized in the light of decreasing La/Nb, and Sr isotope ratios. The orogenic volcanism of Tunisia was followed, after a quiescence period, by the anorogenic basalt of Nefza and Mogods (ca. 8.4 to 5.0 Ma; *Bellon*, 1981). In the Maghreb area, *Maury et al*. (2000) report that the post 16/15 Ma magmatics show a progressive change from calc-alkaline to anorogenic composition, consistent with overall decreasing Sr isotopic values (from 0.720-0.716 to 0.703) and increasing Nd (from 0.5121 to 0.5129). Figure 5 summerizes that, in Sardinia, anorogenic volcanics of Pliocene age (ca. 5.4 to 1.8 Ma) follow the calc-alkaline sequence of the Oligo-Miocene phase. In the late Quaternary (ca. 0.8 to 0.2 Ma), late alkaline-olivine basalts erupt in the Logudoro area of NW Sardinia. In the Magnaghi and

Vavilov seamounts (bathyal plain of Vavilov), are present anorogenic volcanics which show respectively Pliocene and Quaternary age (Fig. 5; *Selli et al*., 1977; *Robin et al*., 1987; *Faggioni et al*., 1995). To the west of the calc-alkaline Aeolian islands and Anchise, Aceste seamount shows anorogenic affinity.

Various authors (see, e.g., *Ellam et al*., 1989; *Gasperini et al*. 2002; *Cadoux et al*., 2005; *De Astis et al*., 2006; *Rosenbaum et al*., 2008; *Trua et al*., 2004, 2010) consider that orogenic volcanics of the Aeolian arc and Smt. Marsili, and Mt. Vesuvius and Palmarola island (Neapolitan area) show compositional features which are slightly transitional between the orogenic and anorogenic series. Cases of concomitant orogenic and anorogenic magmas have been pointed out around the Mediterranean realm (e.g., North Anatolia, *Wilson et al*., 1997; East Anatolia, Nemrut volcano, *Notsu et al*., 1995, *Yilmaz et al*. 1998; East Rhodopes, *Marchev et al*., 1998). *Rehault et alii* (1984) and *Rollet* et alii (2002, Fig. 10) report 18.5, 17.0 Ma for anorogenic volcanics of Ligurian sea-floor (Fig. 3; site 25) which suggests temporal overlap with Sardinia-Provence oceanic spreading. Considering an area wider than the Mediterranean-Tyrrhenian area, anorogenic volcanics followed in time the Neogene to Quaternary orogenic products of the Carpathian-Pannonian and Aegean-west Anatolian subduction zones (*Wilson and Downes*, 1991; *Seghedi et al*., 2005; *Agostini et al*., 2007; *Harangi and Lenkey*, 2007). Localized anorogenic volcanics of late Cretaceous to Quaternary age are found, in the absence of orogenic products, in numerous areas bordering the Mediterranean-Tyrrhenian area, such as Sicily, Sicily channel, Adria foreland, northeastern Italy, Massif Central, Rhine graben, Catalan volcanic zone, southern Spanish Meseta, north Africa (*Cebria and Lopez-Ruiz*; 1995; *Wilson and Bianchini*, 1999; *Azzouni –Sekkal et al*., 2007; *Beccaluva et al*., 2007; *Lustrino and Wilson*, 2007). “Finger-like” diapirism of asthenospheric mantle with HIMU (high U/Pb) characteristics (*Wilson and Bianchini*, 1999; *Lustrino and Wilson*, 2007) is thought to be linked to the anorogenic volcanics of the Mediterranean-Tyrrhenian and its large peripheral areas. The “finger-like” asthenospheric diapirism is most apparent in the Tyrrhenian oceanic opening (Fig. 7), where the likely source of tholeiitic magma occurs at the shallow asthenosphere-lithosphere boundary.

References for the Supplementary Material:

Amorosi A., Coccioni R., Tateo F., 1994. The volcaniclastic bodies in the lower Miocene Bisciaro Formation (Umbria-Marche Apennines, central Italy); in: Miocene stratigraphy of Italy and adjacent regions. Giornale di Geologia, 56/1, 33-46.

Amorosi A., Ricci Lucchi F., Tateo F., 1995. The lower Miocene siliceous zone: a marker in the paleogeographic evolution of the northern Apennines. Palaeogeography., Palaeoclimatology, Palaeoecology, 118 (1-2), 131-149.

Anelli L., Gorza M., Pieri M., Riva M., 1994. Surface well data in the northern Apennines (Italy). Memorie Societa’ Geologica Italiana, 48, 461-471.

Argnani A., Marani M., Savelli C., Galassi B., (1995). Migrazione del vulcanismo di arco cenozoico nel contesto geodinamico intraorogenico del Mar Tirreno meridionale: un riesame. Scritti e Documenti dell'Accademia Nazionale delle Scienze, 14: 377-396.

Argnani A. and C. Savelli (1999), Cenozoic volcanism and tectonics in the southern Tyrrhenian sea: space-time distribution and geodynamic significance, *J. of Geodynamics*, *27*, 409-432.

Argnani A. and C. Savelli (2001), Magmatic signature of episodic back-arc rifting in the southern Tyrrhenian Sea, *Mem. Mus. natn. Hist. nat., 186*, 735-754.

Balogh K., Delle Rose M., Guerrera F., Ravasc-Baranyai L. and F. Veneri (1993), New data concerning the inframiocenic "Bisciaro volcanoclastic event" (Umbria-Marche Apennines) and comparison with similar occurrences, *Giornale di Geologia, 55/2***,** 83-104.

Balogh K., Cassola P., Massimo P. and D. Puglisi (2001), Petrographic, geochemical and radiometric data on Tertiary volcano-arenitic beds from the Sicilian Maghrebian chain: volcanic sources and geodynamic implications, *Geologica Carpathica, 52/1*, 15-21.

Barruol G. and M. Granet (2002), A Tertiary asthenospheric flow beneath the southern French Massif Central indicated by upper mantle seismic anisotropy and related to the west Mediterranean extension, *Earth and Planetary Science Letters, 202*, 31-47.

Baruffini, L., Lottaroli, F. and S. Torricelli S. (2002), Integrated high-resolution stratigraphy of the lower Oligocene Tusa tuffite formation in the Calabro-Lucano area and Sicily (southern Italy), *Rivista Italiana di Paleontologia e Stratigrafia, 108(3)*, 457-477.

Beccaluva, L., Deriu, M., Macciotta, G., Savelli, C. and G. Venturelli (1977), Geochronology and magmatic character of Pliocene-Pleistocene volcanism in Sardinia (Italy), *Bulletin Volcanologique 40/3*, 1–16.
Beccaluva L., Coltorti M., Galassi B., Macciotta G. and F. Siena (1994), The Cenozoic calc-alkaline magmatism of the western Mediterranean and its geodynamic significance. Bollettino. Geofisica Teorica e Applicata, 36/141-144, 293-308.
Belanteur O., Bellon H., Maury R.C. and 5 others (1995), Le magmatisme miocene de l'Est Algerois: geologie, geochimie et geochronologie ^{40}K-^{40}Ar, *Comptes Rendus Academie des Sciences, Paris*, *321*, 489-496.
Bell K., Castorina F Rosatelli G Stoppa F. (2006), Plume activity, magmatism, and the geodynamic evolution of the central Mediterranean, *Annals of Geophysics*, *Suppl. to vol. 49/1*, 357-371.
Bellon H. (1981), Chronologie radiometrique (K-Ar) des manifestations magmatiques autour de la Mediterranee occidentale entre 33 et 1 Ma. In Wezel F.C. (ed.), Sedimentary basins of Mediterranean margins. *CNR Italian Project of Oceanography, Tecnoprint, Bologna*, 341-360.
Bellon H., Bordet P., and C. Montenat (1983), Chronologie du magmatisme neogene des Cordilleres betiques (Espagna meridionale), *Bulletin Societe' Geologique de France, 25*, 205-217.
Benito R., Lopez-Ruiz J., Cebria J.M., Hertogen J., Doblas M., Oyarzun R. and D. Demaiffe (1999), Sr and O isotope constraints on source and crustal contamination in the high-K calc-alkaline and shoshonitic Neogene volcanic rocks of SE Spain, *Lithos, 46*, 733-802.
Boccaletti M., Elter P., and G. Guazzone (1971), Plate tectonic models for the development of the Western Alps and Northern Apennines. *Nature* 234:108–111.
Borsetti A.M., Cati F., Mezzetti R., Savelli C. Toni, G. (1984), Le intercalazioni vulcanoclastiche nei sedimenti oligo-miocenici dell'Appennino settentrionale e centrale (dati petrologici, K/Ar e micropaleontologici), *Giorn. Geol., 45/2*, 159-198.
Cadoux A., Pinti D. L., Aznar C., Chiesa S. and P.-Y.Gillot, (2005), New chronological and geochemical constraints on the genesis and geological evolution of Ponza and Palmarola Volcanic Islands (Tyrrhenian Sea, Italy), *Lithos 81*, 121– 151.
Campos Venuti M., Pini, G. and A. Tateo, F. (1997), Oligocene ash-layer in an Epiligurian Unit of Northern Apennines near Bologna (Italy); how far was the volcano? *Giorn. Geol. 59*, 169-192.
Carminati E., Wortel M.J.R., W. Spakman and R. Sabatini (1998a), The role of slab detachment processes in the opening of central-western Mediterranena basins: some geological and geophysical evidence, *Earth Planetary. Scie. Lett., 160*, 651-665.
Carminati E., Wortel M.J.R., Meijer P.Th. and R. Sabadini (1998b), The two-stage opening of the western–central Mediterranean basins: a forward modeling test to a new evolutionary model, *Earth and Planetary Science Letters, 160*, 667–679.
Cavazza W., Roure F.M., and P.A. Ziegler (2004), The Mediterranean area and the surrounding regions: active processes, remnants of former Tethyan oceans and related thrust belts, in The TRANSMED atlas, edited by Cavazza W., Roure F.M., Spakman W., Stampfli G.M., and P.A. Ziegler, *Springer-Verlag, Berlin Heidelberg New York,* pp. 24-27.
Chiarabba C., De Gori P. and F. Speranza (2008), The southern Tyrrhenian subduction zone: Deep geometry, magmatism and Plio-Pleistocene evolution, *Earth and Planet. Science Lett., 268*, 408–423.
Cibin U., Tateo, F., Catanzariti, R., Martelli, L. Rio D. (1998), Composition, origin, and age of lower Oligocene andesitic volcanism in the Northern Apennines; the volcaniclastic beds of the Ranzano Formation, *Boll. Soc. Geol. It. 117/3*, 569-591.
Cibin, U., Spadafora, E., Zuffa, G G., Castellarin, A. (2001), Continental collision history from arenites of episutural basins in the Northern Apennines, Italy, *Geological Society of America Bulletin*, *113*, 4-19.
Civetta L., Orsi G., Scandone P. and R. Pece (1978), Eastward migration of the Tuscan anatectic magmatism due to anticlockwise rotation of the Apennines, *Nature, 276*, 604-606.
Cocchi, L., Caratori Tontini F., Muccini F., Marani M. P., Bortoluzzi, G., Carmisciano, C., 2009. Chronology of the transition from a spreading ridge to an accretional seamount in the Marsili backarc basin (Tyrrhenian Sea). Terra Nova, 21, 369–374.
Coccioni R., Guerrera F. and F. Veneri (1988), Segnalazione di un intervallo piroclastico (Mega –P) di notevole spessore nel Bisciaro inframiocenico di Arcevia (Appennino marchigiano), *Boll. Soc. Geol. It., 107*, 25-32.
Conte A.M. (1997), Petrology and geochemistry of Tertiary calcalkaline magmatic rocks from the Sarroch district (Sardinia, Italy), *Periodico Miner., 66*, 63-100.
Conticelli S., Carlson R.W., Widom E. and G. Serri (2007), Chemical and isotopic composition (Os, Pb, Nd, and Sr) of Neogene to Quaternary calc-alkalic, shoshonitic and ultrapotassic mafic rocks from the Italian peninsula: Inferences on the nature of their mantle sources, *in* Beccaluva L., Bianchini G., and Wilson M., eds., Cenozoic Volcanism in the Mediterranean Area*: GSA, Special Paper 418*, 171-202.
Conticelli S., Guarnieri L., Farinelli A., Mattei M., Avanzinelli R., Bianchini G., Boari E., Tommasini S., Tiepolo M., Prelević D., Venturelli G. (2009), Trace elements and Sr–Nd–Pb isotopes of K-rich, shoshonitic, and calc-al-

kaline magmatism of the Western Mediterranean Region: Genesis of ultrapotassic to calc-alkaline magmatic associations in a post-collisional geodynamic setting. *Lithos* 107, 68–92.

Coulon C., Demant A., and H. Bellon (1974), Premieres datations par la methode K/Ar de quelques laves Cenozoiques et Quaternaires de Sardaigne nord-occidentale, *Tectonophysics, 22*, 41-57.

Coulon C. and C. Dupuy (1975), Evolution spatiale des caracteres chimiques du volcanism andesitique de la Sardaigne, Italie, *Earth Planet. Sci. Letters, 5*, 170-176.

Crisci G.M., Critelli S. and R. De Rosa (1988), Vulcanismo sinsedimentario nella successione terrigena della formazione di San Mauro (Miocene inferiore, Unità del Cilento), Appennino meridionale, *Mineralogica Petrographica Acta, 31*, 159-178.

Critelli S., De Rosa R., Sonnino M. and G.G. Zuffa (1990), Significato dei depositi vulcanoclastici della Formazione delle Tufiti di Tusa (Miocene inferiore, Lucania meridionale), *Boll. Soc. Geol. It., 109*, 743-762.

Critelli, S. and Le Pera E. (1990), Litostratigrafia e composizione della Formazione di Pollica (Cilento, Appennino meridionale), *Boll. Soc. Geol. It*., 109,511-536.

De Astis, G., P. D. Kempton, and A. Peccerillo (2006), Trace element and isotopic variations from Mt. Vulture to Campanian volcanoes: Constraints for slab detachment and mantle inflow beneath southern Italy, Contrib. Mineral. Petrol., 151, 331 – 351, doi:10.1007/s00410-006-0062-y.

De Capoa P., Di Staso A., Guerrera F., Perrone V., Tramontana M. and M.N. Zaghloul (2002), The lower Miocene volcaniclastic sedimentation in the Sicilian sector of the Maghrebian Flysch Basin: geodynamic implications, *Geodinamica Acta*, *15*, 141-157.

Delle Rose M., Guerrera F., Renzulli A., Ravasc-Baranyai L. and Serrano F. **(**1994a), Stratigrafia e petrografia delle Marne di Vicchio (Unità Tettonica Cervarola) dell'alta Val Tiberina (Appennino Tosco-Romagnolo)**,** *Boll. Soc. Geol. It,. 113*, 675-708.

Delle Rose M., Guerrera F., Renzulli A. and Serrano F. (1994b), Stratigraphic and volcaniclastic events in the Vicchio Marls Auctorum (Cervarola Tectonic Unit) along the Monte Fatucchio section (northern Apennines, Italy), *Giorn. Geol., 56/1*, 97-114.

Deriu M., (1962), Stratigrafia, cronologia e caratteri petrochimici delle vulcaniti "oligoceniche" in Sardegna, *Mem. Soc. Geol. It., 3*, 675-706.

Di Battistini G., Toscani L., Iaccarino S. and I. Villa (1987), K/Ar ages and geological setting of calcalkaline volcanic rocks from Sierra de Gata, SE Spain, *Neues Jahrbuch Mineralogie, Monatshefte H8*, 23-38.

Doblas M., Lopez-Ruiz J. and J.-M. Cebria' (2007), Cenozoic evolution of the Alboran domain: A review of the tectonomagmatic models. *GSA, Special Paper 418*, 303-320.

Doglioni C. (1992), Main differences between thrust belts, *Terra Nova, 4*, 152-164.

Doglioni C. (1995), Geological remarks on the relationships between extension and convergent geodynamic settings, *Tectonophysics, 252*, 253-267.

Doglioni C., Mongelli F. and G.P. Pialli (1998), Boudinage of the Alpine belt in the Apenninic back-arc. *Mem. Soc. Geol. It., 52*, 457 – 468.

Doglioni C., Gueguen E., Harabaglia P. and F. Mongelli (1999a), On the origin of west-directed subduction zones and applications to the western Mediterranean. In: Durand B., Jolivet L., Horvath F. and Seranne M. (eds.), The Mediterranean Basins: Tertiary Extension within the Alpine Orogen. *Geological Society London, Special Publication, 156*, 541-561.

Doglioni C., Harabaglia P., Merlini S., Mongelli F., Peccerillo A. and C. Piromallo (1999b), Orogens and slabs vs their direction of subduction, *Earth Science Reviews, 45*, 167-208.

Doglioni, C., Innocenti, F., Morellato, C., Procaccianti, D., and D. Scrocca (2004), On the Tyrrhenian Sea opening, *Memorie Descrittive della Carta Geologica d'Italia, 64*, 147–164.

Dostal J., Coulon C., and C. Dupuy (1982), Cainozoic andesitic rocks of Sardinia (Italy), in: Andesites, orogenic andesites and related rocks, ed. R.S. Thorpe, John Wiley & Sons, Chichester, United Kingdom, 353-370.

Downes, H., Thirlwall, M.F. and S.C. Trayhorn (2001), Miocene subduction-related magmatism in southern Sardinia: Sr-Nd- and oxygen isotopic evidence for mantle source enrichment, *Journal of Volcanology and Geothermal Research, 106*, 1-21.

Duggen S., Hoernle K., van den Bogaard P., Harris C. (2004), Magmatic evolution of the Alboran region: The role of subduction in forming the western Mediterranean and causing the Messinian Salinity Crisis, *Earth Planet. Scie. Lett. 218*, 91-108.

El Azzouzi M., Bernard-Griffith J., Bellon H., Maury R.C., and 4 others, (1999), Evolution des sources du volcanism marocain au cours du Neogene, *Comptes Rendu Academie des Sciences, Paris (terre e planetes), 329*, 95-102.

El Bakkali, S., A. Gourgaud, J. L. Bourdier, and N. Gundogdu (1998), Post-collision neogene volcanism of the Eastern Rif (Morocco): Magmatic evolution through time, Lithos, 45, 523 – 543.

Ellam, R. M., M. A. Menzies, C. J. Hawkesworth, and N. W. Rogers (1989), The volcanism of southern Italy: Role of subduction and the relationship between potassic and sodic alkaline magmatism, *J. Geophys. Res., 94*, 4589 – 4601.

Faccenna C., Piromallo C., Crespo-Blanc A., Jolivet L. and F. Rossetti (2004), Lateral slab deformation and the origin of the western Mediterranean arcs, *Tectonics, 23*, 21 pages, TC1012, doi:10.1029/2002TC001488.

Faccenna C., Funiciello F., Civetta L., D'Antonio M., Moroni M. and C. Piromallo (2007), Slab disruption, mantle circulation, and the opening of the Tyrrhenian basins, *in* Beccaluva L., Bianchini G., and Wilson M., eds., Cenozoic Volcanism in the Mediterranean Area*: GSA, Special Paper 418*, 153-169.

Faggioni O., Pinna E., Savelli C. and A.A. Schreider (1995), Geomagnetism and age study of Tyrrhenian seamounts*, Geophys. J. Intern., 123,* 915-930.

Faugeres J.C., Broquet P., Duée G. and P. Imbert (1992), Episodes volcano-sedimentaires et paleo-courants dans le Numidien externe de Sicile: les tuffites et contourites de Karsa, *Comptes Rendus Academie des Sciences Paris, 315*, 479-486.

Fourcade, S., Capdevila, R., Aziouz, O., Martineau, F., (2001), The origin and geodynamic significance of the Alpine cordierite-bearing granitoids of northern Algeria. A combined petrological, mineralogical, geochemical and isotopic (O, H, Sr, Nd) study, *Lithos*, *57/2-3*, 187-216.

Francalanci L. and P. Manetti (1994), Geodynamic models of the southern Tyrrhenian region: constraints from the petrology and geochemistry of the Aeolian volcanic rocks. *Bollettino Geofisica Teorica e Appllicata*, 36/141-144, 283-292.

Franciosi L., Lustrino M., Melluso M. and M. D'Antonio (2003), Geochemical characteristics and mantle sources of the Oligo-Miocene primitive basalts from Sardinia: the role of subduction components, *Ofioliti, 28/2*, 105-114.

Gasperini, D., J. Blichert-Toft, D. Bosch, A. Del Moro, P. Macera, and F. Albare`de (2002), Upwelling of deep mantle material through a plate window: Evidence from the geochemistry of Italian basaltic volcanics, J. Geophys. Res., 107(B12), 2367, doi:10.1029/2001JB000418.

Gattacceca, J., Deino, A., Rizzo, R., Jones, D.S., Henry, B., Beaudoin, B. and F. Vadeboin (2007), Miocene rotation of Sardinia: New paleomagnetic and geochronological constraints and geodynamic implications, *Earth and Planetary Science Letters, 258/3-4*, 359-377.

Gill, R.C.O., Aparicio A., El Azzouzi M., Hernandez J., Thirlwalla, M.F., Bourgoise J. and Marriner G.F. (2004), Depleted arc volcanism in the Alboran Sea and shoshonitic volcanism in Morocco: geochemical and isotopic constraints on Neogene tectonic processes, *Lithos, 78*, 363– 388.

Gueguen E., Doglioni C. and M. Fernandez (1997), Lithospheric boudinage in the western Mediterranean back-arc basin, *Terra Nova, 9*, 184-187.

Gueguen, E., Doglioni, C. &. Fernandez M. On the post-25 Ma geodynamic evolution of the Western Mediterranean. Tectonophysics*,* **298**, 259-269 (1998).

Guerrera F., Tonelli G. Veneri F. (1986), Caratteri lito-sedimentologici e mineralogico-petrografici di vulcanoclastiti mioceniche presenti nella Successione Umbro-Marchigiana**,** *Boll. Soc. Geol. It., 105/3-4*, 307-325.

Guerrera F., Martin-Algarra A. and V. Perrone (1993), Late Oligocene-Miocene syn-late-orogenic successions in Western and Central Mediterranean Chains from the Betic Cordillera to the Southern Apennines, *Terra Nova, 5*, 525-544.

Guerrera F., Mattioli M., Puglisi D., Renzulli A., Santi P., Veneri F., Assorgia A. and K. Balogh (1998), An overview of the upper Oligocene-lower Miocene volcanogenic sediments in the Western Mediterranean and their possible source areas, *Romanian Journal of Stratigraphy, 78*, 43-56.

Guerrera F., Mattioli M., Serrano F., Tramontana M. and G. Raffaelli **(**2004), Stratigraphy of the Miocene syn-rift volcano-sedimentary succession in a sector of the central-southern Sardinia trough (Italy), *Geologica Carpathica – International Geological Journal, 55/1,* 51-63.

Guerrera F., Martin-Martin M., Perrone V. and M. Tramontana (2005), Tectono-sedimentary evolution of the southern branch of the Western Tethys (Magrebian Flysch Basin and Lucanian Ocean), *Terra Nova, 17*, 358-367.

Guerrera F., Tramontana M., Donatelli U. and F. Serrano (2012), Space/time tectono-sedimentary evolution of the Umbria-Romagna-Marche Miocene Basin (Northern Apennines, Italy): a foredeep model, *Swiss J Geosci*, 105:325–341. DOI 10.1007/s00015-012-0118-0

Gutscher M.-A., Malod J., Rehault J.-P., Contrucci I., Klingelhoefer F., Mendes-Victor L. and W. Spakman (2002), Evidence for active subduction beneath Gibraltar, *Geology, 30*, 1071–1074.

Hawkesworth, C., S. Turner, K. Gallagher, A. Hunter, T. Bradshaw, and N. Rogers (1995), Calc-alkaline magmatism, lithospheric thinning and extension in the Basin and Range, *J. Geophys. Res.*, 100(B6), 10,271–10,286.

Heymes, T. F., Bouillin J.P., Pecher A., Monie P., R. Compagnoni. 2008. Middle Oligocene extension in the Mediterranean Calabro-Peloritan belt (Southern Italy). Insights from the Aspromonte nappes-pile. Tectonics, doi:10.1029/2007TC002157.

Hoernle K., v.d. Bogaard P., Duggen S., Mocek B. and D. Garbe-Schoenberg (1999), Evidence for Miocene subduction beneath the Alboran Sea: 40Ar/39 Ar dating and geochemistry of volcanic rocks from Holes 977A and 978A, in: R. Zahn, M.C. Comas, A. Klaus (Eds.), *Proc. ODP Sci. Results, 161,* 357-373.

Hernandez J., De Larouziere F. D., Bolze J. and P.Bordet (1987), Le magmatisme Neogene betico-rifain et le couloir de decrochement trans-Alboran. *Bulletin Societe' Geologique de France, (8) III/2*, 257-267.

Hooper, P. R., Bailey, D. G. and G. A. M. Holder (1995), Tertiary calc-alkaline magmatism associated with lithospheric extension in the Pacific Northwest, J. Geophys. Res., 100(B6), 10,303–10,319.

Kastens, K., Mascle, J., Auroux, C., Bonatti E., Broglia C., Channell J.E.T, Curzi P., Emeis K.C., Glason G., Hasegawa S., Hieke W., Mascle G., Mccoy F., Mckenzie, Mendelson, J., Mueller, C., Rehault, J-P., Robertson, A., Sartori, R., Sprovieri, R. & Torii M. ODP Leg 107 in the Tyrrhenian Sea: Insigths into passive margin and back-arc basin evolution, *Geological Society of America Bulletin,* **100**, 1140-1156 (1988).

Keller J.V.A., Minelli, G. and G. Pialli (1994), Anatomy of late orogenic extension: the northern Apennines case, *Tectonophysics, 238*, 275–294.

International Stratigraphic Chart (2009), International Commission on Stratigraphy (ICS–IUGS; www.stratigraphy.org/).

Lavecchia G. and F. Stoppa (1990), The Tyrrhenian zone: a case of lithosphere extension control of intra-continental magmatism, *Earth Planetary Science Letters, 99*, 336-350.

Lavecchia G. and F. Stoppa (1996), The tectonic significance of Italian magmatism: an alternative view to the popular interpretation, *Terra Nova*, *8*, 435-446.

Le Pera E. and G. Ventura (1994), Geochemical features of the Gorgoglione flysch volcaniclastic detritus (Lucanian Apennines, southern italy), Miner. Petrogr. Acta, 37, 379-391.

Lonergan, L. and N. White (1997), Origin of the Betic-Rif mountain belt, *Tectonics, 16*, 504-522.

Louni-Hacini A., Bellon H., Maury R.C., and 5 others, (1995), Datation 40K-40Ar de la transition du volcanisme calco-alcalin au volcanisme alcalin en Oranie au Miocene superieur, *Comptes Rendus Academie des Sciences, Paris, 321*, 975-982.

Lustrino M., Morra V., L. Melluso, 2004. The Cenozoic igneous activity of Sardinia. *Periodico di Mineralogia, 73*, 105-134.

Lustrino M. and M. Wilson (2007), The circum-Mediterranean anorogenic Cenozoic igneous province, *Earth Science Reviews, 81*, 1-65.

Maate A., Martin Perez J.A., Martin Algarra A., Serrano F., Aguado R., Martin Martin M. and Kh. El Hajaji (1995), Le Burdigalien inférieur de Boujarrah (Rif septentrional, Maroc) et la signification paléotectonique des séries miocènes transgressives sur les zones internes bético-rifaines, *Comptes Rendus Academie des Sciences Paris, 320/Série Iia*, 15-22.

Malinverno A. and W.B.F. Ryan (1986), Extension in the Tyrrhenian Sea and shortening in the Apennines as a result of arc migration driven by sinking of the lithosphere, *Tectonics, 5*, 227-245.

Mantovani E., Albarello D., Babbucci D., Tamburelli C., Viti M., (2002). Trench-arc-Backarc systems in the Mediterranean area: examples of extrusion tectonics. *Journal of Virtual Explorer, Vol.8*, 125-141.

Marani M.P. and Trua T. (2002), Thermal constriction and slab tearing at the origin of a superinflated spreading ridge: Marsili volcano (Tyrrhenian Sea), *J. Geophys. Res., 107/ B9*, 2188, doi:10.1029/2001JB000285.

Marti' J., Mitjavila J., Roca E., Aparicio A., (1992), Cenozoic magmatism of the Valencia trough (western Mediterranean): relationship between structural evolution and volcanism, *Tectonophysics*, *203,* 145-165.

Martin, A. K. (2006), Oppositely directed pairs of propagating rifts in back-arc basins: Double saloon door seafloor spreading during subduction rollback, *Tectonics, 25*, TC3008, doi:10.1029/2005TC001885.

Mattioli M., Guerrera F., Tramontana M., Raffaelli G. D'Atri M., (2000) High-Mg Tertiary basalts in Southern Sardinia (Italy), *Earth Planetary Science Letters, 179*, 1-7.

Mattioli M., Di Battistini G. and G. Zanzucchi (2002), Petrology, geochemistry and age of the volcanic clasts from the Canetolo Unit (Northern Apennines, Italy), *Boll. Soc. Geol. It. Special Volume 1 (Geological and geodynamic evolution of the Apennines)*, 399-416.

Mascle G.H., Tricard P., Torelli L., Bouillin J-P., Rolfo F., Lapierre H., Monie' P., Depardon S., Mascle J., Peis D. (2001), Evolution of the Sardinia Channel (Western Mediterranean): new constraints from a diving survey on Cornacya seamount off SE Sardinia, *Marine Geology, 179*, 179-202.

Maury C. R., Fourcade, S., Coulon, C., El Azzouzi, M.; Bellon, H., Coutelle, A., Ouabadi, A., Semroud, B., Megartsi, M., Cotten, J., Belanteur, O., Louni-Hacini, A., Piqué, A., Capdevila, R. Hernandez and J. P. Réhault (2000), Post-collisional Neogene magmatism of the Mediterranean Maghreb margin: a consequence of slab breakoff, *C.R. Acad. Sci. Paris*, *331,* 159-173.

Mezzetti R., and R. Olivieri (1964), Intercalazioni cineritiche di eta' oligocenica e miocenica nell'Appennino centro-settentrionale, (Volcanic ash layers of Oligo-Miocene age of central-northern Apennines; with english abstract) *Miner. Petrogr. Acta, 10*, 129-147.

Monie', P., Maluski, H., Saadallah, A. and R., Caby (1995), New 39Ar-40Ar ages of Hercynian and Alpine thermometamorphic events in Grande Kabylie (Algeria), *Tectonophysics, 152*, 53–69.

Montigny, R., J.B. Edel and R. Thuizat (1981), Oligo-Miocene rotation of Sardinia: K-Ar ages and paleomagnetic data of Tertiary volcanics, *Earth Planet. Sci. Letters* 54, 261–271.

Morra V., Secchi, F. and A. Assorgia (1994), Petrogenetic significance of peralkaline rocks from Cenozoic calc-alkaline volcanism from SW Sardinia, Italy, *Chem. Geol., 118/1-4*, 109-142.

Morra, V., Secchi, F.A.G., Melluso, L. and L. Franciosi (1997), High-Mg subduction-related Tertiary basalts in Sardinia, Italy, *Lithos, 40*, 69-91.

Nicolosi I., Speranza F., and M. Chiappini (2006), Ultrafast oceanic spreading of the Marsili Basin, southern Tyrrhenian Sea: Evidence from magnetic anomaly analysis, *Geology, 34/9,* 717-720.

Ottaviani-Spella M.-M., Girard M., Rochette P., Cheilletz A. Thinon M. (2001), Le volcanisme acide Burdigalien du sud de la Corse: petrologie, datation K-Ar, paleomagnetism, *Comptes Rendus Academie des Sciences Paris, 333*, 113-120.

Patacca E., Sartori R. and P. Scandone (1990), Tyrrhenian basin and Apenninic arcs. Kinematic relations since late Tortonian times, *Mem. Soc. Geol. It., 45*, 425-451.

Peccerillo A. (1999), Multiple mantle metasomatism in centralsouthern Italy: geochemical effects, timing and geodynamic implications, *Geology, 27/4*, 315-318.

Perrone V. (1987), I depositi miocenici della dorsale di Monte Soprano (Appennino campano): segnalazione di vulcanismo andesitico e nuova interpretazione, *Boll. Soc. Geol. It., 106*, 3-12.

Prelevic' D. and S.F. Foley (2007), Accretion of arc-oceanic lithospheric mantle in the Mediterranean: Evidence from extremely high-Mg olivines and Cr-rich spinel inclusions in lamproites, *Earth and Planet. Science Lett., 256*, 120–135.

Rehault J.P., Boillot G. and A. Mauffret (1984), The western Mediterranean basin geological evolution, *Marine Geology, 55*, 447-477.

Riviere M., Bouillin, J.P., Courtois, C., Gelard, J.P. and J.F. Raoult (1977), Etude mineralogique et geochimique des tuffites decouvertes dans l'Oligo-Miocene Kabyle (Grande Kabylie- Algerie). Compairason avec les tuffites de la region de Malaga (Espagne), *Bulletin Societe Geologique de France, 19*, 1171–1177.

Robin, C., Colantoni, P., Gennesseaux, M. and J.P., Rehault (1987), Vavilov seamount: a mild alkaline Quaternary volcano in the Tyrrhenian basin, *Marine Geol., 78*, 125-136.

Rollet N., J. Déverchère, M. Beslier, P. Guennoc, J. Réhault, M. and C. Sosson (2002), Truffert, Back arc extension, tectonic inheritance, and volcanism in the Ligurian Sea, Western Mediterranean, *Tectonics, 21/3,* 1015, doi:10.1029/2001TC900027.

Rosenbaum G., Lister G.S. and C. Duboz (2002), Reconstruction of the tectonic evolution of the Western Mediterranean since the Oligocene, *J. Virtual Explorer, 8*, 107-126.

Rosenbaum, G., M. Gasparon, F. P. Lucente, A. Peccerillo, and M. S. Miller (2008), Kinematics of slab tear faults during subduction segmentation and implications for Italian magmatism, *Tectonics*, *27*, TC2008, doi:10.1029/2007TC002143.

Rossi P., Guennoc P., Rehault J.P. and 9 others (1998), Importance du volcanisme calco-alcalin Miocene sur la marge SO de la Corse (campagne Marco), *Comptes Rendue Academie des Sciences, Paris, 327*, 369-376.

Ryan W.B.F., Hsu K.J., Honnorez J., Weibel M., Cann J.R., Ferrara G., Bigazzi G., Bonadonna F.P. and O. Giuliani (1972), Petrology and geochemistry of the Valencia Trough volcanic rocks, *Init. Rep. Deep Sea Drilling Project, 13*, 767-773.

Sartori R., Torelli L., Zitellini N., Carrara G., Magaldia M. and P. Mussoni (2004), Crustal features along a W–E Tyrrhenian transect from Sardinia to Campania margins (Central Mediterranean). *Tectonophysics, 383*, 171–192.

Savelli C., Beccaluva L., Deriu M., Macciotta G. and L. Maccioni (1979), K/Ar geochronology and evolution of the Tertiary "calcalkalic" volcanism of Sardinia (Italy), *J. of Volcanology and Geothermal Research, 5*, 257-269.

Savelli C. (1984), Evoluzione del vulcanismo cenozoico (da 30 Ma al presente) nel Mar Tirreno e nelle aree circostanti: ipotesi geocronologica sulle fasi dell'espansione oceanica, (Cenozoic volcanism evolution in the Tyrrhenian region: geochronological hypothesis on the phases of oceanic accretion, English abstract), *Mem. Soc. Geologica It., 27*, 111-119.

Savelli, C. & Gasparotto, G., (1994), Calcalkaline magmatism and rifting of the deep-water volcano of Marsili (Aeolian back-arc, Tyrrhenian sea), *Marine Geology, 119*, 137–147.

Savelli, C., (2000), Subduction-related episodes of K-alkaline magmatism (15–0 Ma) and geodynamic implications in the north Tyrrhenian-central Italy region: a review. *Journal of Geodynamics, 30*, 575–591.

Savelli, C. (2002), Time-space distribution of magmatic activity in the western Mediterranean and peripheral orogens during the past 30 Ma (a stimulus to geodynamic considerations*)*, *J. of Geodynamics*, *34*, 99-126.

Savelli, C., 2005. Post-Eocene calcalkaline activity and basin opening in the western and central Mediterranean region: implications for magma source metasomatism linked to Hercynian orogeny. *Boll. Soc. Geol. It., Special Volume n. 4*, 119-125.

Savelli, C., 2011. The west Mediterranean-Tyrrhenian since the Oligocene: Post-collision development of calc-alkaline volcanism and basin formation. 7° Forum of Earth Sciences, Torino, 101474/Epitome.04.0542.Geoitalia 2011

Scrocca D., Doglioni C. and E. Carminati (2005), Deep structure of the southern Apennines, Italy: Thin-skinned or thick-skinned? *Tectonics, 24* TC3005, Doi:10.1029/2004tc001634.

Selli, R., Lucchini, F., Rossi, P.L., Savelli, C. and M., Del Monte (1977), Dati geologici, petrochimici e radiometrici sui vulcani centro-tirrenici, *Giorn. Geol., 42/1*, 221-246.

Serri, G., Innocenti F. and P. Manetti (1993), Geochemical and petrological evidence of the subduction of delaminated Adriatic continental lithosphere in the genesis of the Neogene-Quaternary magmatism of central Italy, *Tectonophysics, 223,* 117 – 147.

Spadea P. and R. Carmisciano (1974), Nuovi dati sulle cineriti riodacitiche del Miocene medio della Sicilia centro-meridionale, (New data on mid Miocene rhyodacitic ashes of south-central Sicily), *Boll. Soc. Geol. It., 93*, 1095-1126.

Spakman W. and M.J.R. Wortel (2004), A tomographic view on western Mediterranean Geodynamics, in: W. Cavazza, F.M. Roure, W. Spakman, G.M. Stampfli, P.A. Ziegler (Eds.), *The Transmed Atlas – The Mediterranean Region from Crust to Mantle*, Springer, Berlin, pp. 31–52.

Stewart J. H., (1978), Basin-range structure in western North America: A review. *Geological Society of America Memoirs, 152*, 1-32. doi:10.1130/MEM152-p1

Tateo F. (1993), Intercalazioni vulcano-sedimentarie nella Formazione di Antognola: le sezioni “Rio Nespolo” e “M.Varano” (Oligocene superiore, Appennino parmense), *Mineralogica Petrographica Acta, 36*, 61-79.

Tatsumi, Y., Maruyama, S. and Nohda, S., 1990. Mechanism of backarc opening in the Japan Sea: role of asthenospheric injection. In: M. Kono and B.C. Burchfiel (Editors), Tectonics of Eastern Asia and Western Pacific Continental Margin. Tectonophysics, 181: 299-306.

Torres Roldan R.L., Poli G. And A. Peccerillo (1986), An early Miocene arc-tholeiitic magmatic event from the Alboran Sea - Evidence for precollisional subduction and back-arc crustal extension in the westernmost Mediterranean, *Geologische Rundschau, 75/1*, 219-234.

Trua T., Serri G., Rossi P.L. (2004) Coexistence of IAB-type and OIB-type magmas in the southern Tyrrhenian back-arc basin: evidence from recent seafloor sampling and geodynamic implications *Mem. Descr. Carta Geol. d'It.* XLIV (2004), pp.83-96.

Trua, T., Clocchiatti R., Schiano P., Ottolini L. and M. Marani (2010), The heterogeneous nature of the Southern Tyrrhenian mantle: Evidence from olivine-hosted melt inclusions from back-arc magmas of the Marsili seamount, *Lithos*, *118*, 1-16 doi:10.1016/j.lithos.2010.03.008

Turner S.P., Platt J.P., George R.M.M., Kelley S.P., Pearson D.G. and G.M. Nowell (1999), Magmatism associated with orogenic collapse of the Betic-Alboran Domain, SE Spain, *J. Petrol. 40,* 1011 - 1036.

Vignaroli G., Faccenna C., Jolivet L., Piromallo C. and F. Rossetti (2008), Subduction polarity reversal at the junction between the Western Alps and the Northern Apennines, Italy, *Tectonophys*., *450*, 34-50.

Viti M., Mantovani E., Babbucci D. & C. Tamburelli (2009) Generation of Trench-Arc-Back Arc Systems in The Western Mediterranean Region driven by plate convergence Ital.J.Geosci. (Boll.Soc.Geol.It.), Vol. 128, No. 1, pp. 89-106, 9 figs. (DOI :10.3301/IJG.2009.128.1.89).

Wilson M. and G. Bianchini (1999), Tertiary-Quaternary magmatism within the Mediterranean and surrounding regions. In: Durand B., Jolivet L., Horvath F. and Seranne M. (eds.), The Mediterranean basins: Tertiary extension within the Alpine orogen, *Geological Society Special Publication, 156*, 141-168.
