# Supplementary material for: Fast Episodes of West-Mediterranean-Tyrrhenian Oceanic Opening and Revisited Relations with Tectonic Setting
Source: Sci Rep. 2015 Sep 22;5:14271. doi: 10.1038/srep14271 (PMC4585736; doi:10.1038/srep14271)
Supplement: Supplementary Information S5 [file srep14271-s4.doc]

**Dataset S5.** Site, formation, age and stratigraphic data of calc-alkaline volcanogenic allochthonous sediments from the Apennines and the Maghrebid-Betic region. Key: SIV, stratigraphic interval with volcanogenic sediments (meters); VTT, total thickness of volcanoclastic beds; TVB, thickest volcanogenic bed. Fig. 3: elongated triangles without black rim of red, gray and yellow colour indicate beds of Oligocene-Aquitanian, Burdigalian and Langhian-Tortonian age. References.- [1] Guerrera et al. 1998 and references therein; [2] Guerrera et al. 2005 and references therein; [3] Critelli et al., 1990; [4] Maate et al., 1995; [5] Riviere et al., 1977; [6] Balogh et al. 1993 and references therein; [6a] Critelli et al., 1990a; [7] Spadea et al. 1974; [8] Le Pera et al., 1994; [9] Balogh et al., 2001; [10] Borsetti et al., 1984; [11] Guerrera et al., 1993 and references therein; [11a] Perrone, 1987; [12] de Capoa et al., 2002; [13] Baruffini et al., 2002; [14] Faugères et al., 1992; [15] Delle Rose et al.,1994a and references therein; [16] Crisci et al., 1988; [17] Mattioli et al. 2002; [18] Cibin et al., 1998; [19] Tateo, 1993; [20] Mezzetti et al., 1964; [21] Cibin et al., 2001; [22] Amorosi et al., 1994; [23] Coccioni et al., 1988; [24] Guerrera, 1977; [25] Guerrera et al.,1986; [26] Delle Rose et al., 1994b; [27] Guerrera et al., 2004; [28] Mattioli et al., 2000 Revisited geochemistry data of samples from sites marked by asterisk (*) is shown in *Supplementary dataset S6*.

| SECTOR,  DOMAIN,  SITE (Fig. 3) | FORMATION | AGE | VOLCANOCLASTIC ROCK TYPE | VOLCANIC LAYERS  (type, SIV, VTT, TVB (meters) | SEDIMENTARY PROCESSES | VOLCANIC SOURCE HYPOTH. | MAIN  REFER-ENCE |
| --- | --- | --- | --- | --- | --- | --- | --- |
| Betic Cordillera/ 1 | Viñuela  Group | early  Burdigalian | andesite, rhyolite- rhyodacite fragments | mineral grains and glass-shards; SIV: 45; VTT: 3; TVB: 0.5 | pyroclastic fallout; epiclastic mass-flow (turbiditic) | ---- | [1]  [2] |
| Betic Cordillera/ 2 | Algeciras Flysch | Aquitanian  Burdigalian p.p. | andesite, rhyolite  fragments | felsitic lava and mineral clasts (up to 7% vol.) | epiclastic mass- flow (turbiditic) | ---- | [1] |
| Betic Cordillera/ 3 | Almidar, Río Fardes-Mencal Succ.b (Subbetic) | early  Burdigalian | rhyodacite  fragments | glass-shards and  mineral grains; TVB: 0.25 | Epiclastic mass-flow (turbiditic) | Submarine volcanic emissions |
| Rif / 4 | Sidi Abdeslam –Boujarrah Fm | Early  Burdigalian | andesite, basalt | mainly mineral clasts, SIV: 10; VTT: 0.5; TVB:0.15 | epiclastic mass-flow (turbiditic) | Internal zones | [1]  [4] |
| Rif / 5 | Beni Ider  Flysch | late Aquitanian -early Burdigalian | andesite,  basalt fragments | lava clasts | syn- and/or post-eruptive epiclastic mass-flow (turbiditic) | Internal zones | [1]  [6]  [2] |
| Rif / 6 | Talaa Lakra (Mixed Successions) | late Aquitanian -early Burdigalian | andesite fragments | lava clasts | syn- and/or post-eruptive epiclastic mass-flow (turb.) | Internal zones | [1]  [6] |
| Algerian Tell/ 7, 8 | Oligo-Miocène Kabylia | Burdigalian p.p (19.1±1.0 Ma) | rhyolite | glass-shards; grains of quartz, plagioclase, sanidine, biotite | epiclastic mass-flow (turbiditic) | Western Mediterran | [11]  [5] |
| Sicily/ 9 | Tusa  Tuffites; | early Oligocene | andesite to dacite fragments | lava clasts (80-85% vol.), grain size up to 2.5 mm, SIV: 600; VTT: 200; TVB: 1.5-2 | epiclastic mass-flow (turbiditic) | Mesomedi- terranean Microplate | [2]  [1]  [12, 13] |
| Sicily/ 10 | Troina Sandstones /  Poggio Maria Sandstones | Burdigalian pp. | andesite  fragments /  volcanic glass, andesite-dacite  fragments | up to 40 % volcanic clasts, grain size >2 mm; SIV: 150, VTT: 30-40; TVB: 0’5-1 /  lava and mineral clasts (30-35% vol.), grain size >2 mm; SIV: 200; VTT: 30-35; TVB: 0’5-1,5 | epiclastic mass-flow (turbiditic) | Mesomedit. Microplate | [2]  [6]  [12] |
| Sicily/ 11* | Case Liardo, M. Ugliarella; C. Biticchie | mid Miocene | rhyodacitic glass | shards, biotite; bentonite clays (TVB: 1-1.5); rhyodacitic tuff (TVB: 0.5-1.5) |  | Volcanic arc | [7] |
| Sicily/ 12 | mixed  successions | late Aquitanian early Burdigal. | rhyolite, andesite, basalt fragments | mainly lava clasts, grain size>2 mm, SIV:43; TVB:1 | epiclastic mass-flow (turbiditic) | --- | [1]  [11] |
| Sicily/ 13* | Reitano flysch; mixed  successions | early Oligocene | arenitic beds; andesite & basalt  fragments | lava and mineral clasts,  glass-shards, pumices, SIV:20 | epiclastic mass-flow (turbiditic) | ---- | [9]  [11,14] |
| Calabria-Peloritani Arc / 14 | Stilo-Capo d’Orlando, Paludi | early Oligocene | andesite & basalt  rock fragments; crystals | lava and mineral clasts | epiclastic mass-flow (turbiditic) | Internal  zones | [1]  [2] |
| S. Apennines  / 15 | Calabro-Lucano Liguridi Complex) | late  Oligocene | andesite, basalt, rhyodacite  fragments | Lava and mineral clasts, glass-shards (andesite, qz-andesite tuffites),TVB:1.5 | epiclastic mass-flow (turbiditic) | Active volcanic arc | [15] |
| S. Apennines  / 16 | Saraceno  (Liguridi Complex) | early  Miocene | andesite to dacite  fragments; mineral clasts | lava and mineral clasts (andesite, qz andesite tuff) | epiclastic mass-flow (turbiditic) | Active volcanic arc | [1]  [2]  [3] |
| S. Apennines  / 17 | Tusa Tuffites complex  (Sicilide Complex) | early Oligocene | andesite, basalt,  dacite fragments | lava and mineral clasts (80%vol.), pelites with pumices, glass-shards;SIV:54; VTT:46;TVB:5 | epiclastic mass-flow (turbiditic) and ash turbidites | Sardinia | [11]  [13  [2, 3] |
| S. Apennines  / 18 | Numidian Flysch | late Burdigalian-  -early Langhian | rhyolite | glass-shards, mineral grains, pumices, lava clasts | Pyroclastic fallout | Sardinia | [11] |
| S. Apennines  / 19 | Pollica (Cilento Group) | Burdigalian- | rhyolite, rhyodacite | lava and tuff clasts, TVB: 3 | epiclastic mass-flow (turbiditic) | Sardinia | [6, 6a] |
| S. Apennines  / 20* | San Mauro/  Pollica (Cilento) | Burdigalian | rhyolite, rhyodacite | lava and mineral clasts; SIV: 15-20 | mass-flow short-distance (turbiditic) | Sardinia-  -Corsica | [16]  [11] |
| S. Apennines  / 21 | Mt. Soprano  (Daunia Complex) | late Burdigalian | andesite and/or basalt fragments | 3-15 % lava clasts; mineral grains, glass-shards; TVB: 5 | pyroclastic fall-out, epiclastic mass-flow (turbiditic) | Volcanic Arc (Sardinia) | [11a] |
| S. Apennines  / 22* | Macchialupo; Gorgoglione  (Daunia comp) | Burdigalian p.p.; Miocene | andesite and/or basalt fragments;  rhyolite clasts | lava clasts (70-90 % vol.), SIV: 80; TVB: 4 | epiclastic mass-flow (turbiditic) | Volcanic Arc (Sardinia) | [6]  [8] |
| N. Apennines  Sub-Ligure Domain / 23 [*] | Val Aveto;  Ranzano | Early Oligocene | basalt, andesite, dacite, dacite-rhyol. ignimbrite clasts | lava clasts  (up to > 80 vol. %)  SIV: 490 | epiclastic mass-flow (turbiditic) | Continental arc (near the graben) | [17] [18] |
| N. Apennines  Sub-Ligure Domain / 24* | Petrignacola;  Ranzano | early Oligocene;  32-30 Ma | basalt, andesite, dacite; rhyodacite, ignimbrite clasts | volcanic clasts  (up to > 80 vol. %); SIV: 255 | epiclastic mass-flow (turbiditic) | Continental arc (near the graben) | [17] |
| N. Apennines  Epi-Ligure Domain / 25* | Antognola Ranzano Mongardino | early Oligocene;  32-30 Ma | andesite, dacite; glass-shards (rhyolite-rhyodacite) | lava and mineral clasts; glass-shards; SIV: 15; VTT: 5; TVB: 5 | epiclastic mass-flow (turbiditic) | ---- | [19] [20]  [1]  [21] |
| N. Apennines  Epi-Ligure Domain / 26/* | Tripoli di Contignaco | Aquitanian-Burdigalian p.p.; 22.4 Ma | rhyodacite-dacite clasts | glass-shards (< 90% vol.), mineral clasts (grain size < 0,5 mm), SIV: 15; VTT: 15; TVB: 10 | pyroclastic fallout, and/or epiclastic mass -flow, hyaloclastic | ---- | [6]  [10]  [22] |
| N. Apennines  / 27[*] | Bisciaro  Formation (s.s.) | Burdigalian | andesite –  rhyolite | glass-shards, mineral and lava clasts; SIV: >100; VTT:18; TVB: 6.9 | epiclastic mass-flow (turbiditic) and pyroclastic fallout | Intern. volc. zone (near the graben) | [1] [5] [23] [24, 25] |
| N. Apennines  / 28 | Cervarola | Aquitanian/  Burdigalian | andesite and  rhyolite | mainly glass-shards, mineral grains -vitric tuffs, SIV: 80; VTT: 10; TVB: 6 | epiclastic mass-flow  (turbiditic) | ---- | [15] |
| N. Apennines  / 29* | Vicchio Marls  (Cervarola Unit; Bisciaro form, l.s.) | late Aquitanian -Burdigalian p.p. (lower portion) | rhyolite  -dacite glass | mineral grains, glass-shards and andesitic clasts; SIV: 450; TVB: 0.3 | epiclastic mass-flow  (turbiditic) | internal volcanic zone (near the graben) | [15]  [26] |
| N. Apennines  / 29a* | Laga Formation | late Miocene | rhyolite glass;  plagioclase | VTT: 1.5-3; TVB: 0.75-1 | Volcanoclastic turbidites | idem | [25] |
| W-Sardinia Graben / 30 | Villanovaforru Succession,  Marmilla Basin | Burdigalian | high-Mg basalt (tholeiitic/calc-alkaline type) | large amount of reworked volcanic detritus, SIV: 640; VTT>10; TVB: 0,6 | epiclastic mass-flow (turbiditic), pyroclastic fall. | close to the source | [27]  [28] |
